# Transcriptional profiling of differentially vulnerable motor neurons at pre-symptomatic stage in the *Smn*^*2b/-*^ mouse model of spinal muscular atrophy

**DOI:** 10.1186/s40478-015-0231-1

**Published:** 2015-09-15

**Authors:** Lyndsay M. Murray, Ariane Beauvais, Sabrina Gibeault, Natalie L. Courtney, Rashmi Kothary

**Affiliations:** Regenerative Medicine Program, Ottawa Hospital Research Institute, Ottawa, ON K1H 8 L6 Canada; Centre for Integrative Physiology, University of Edinburgh, Edinburgh, EH8 9XD UK; Euan McDonald Centre for Motor Neuron Disease Research, University of Edinburgh, Edinburgh, EH8 9XD UK; Department of Cellular and Molecular Medicine, University of Ottawa, Ottawa, ON K1H 8 M5 Canada; Department of Medicine, University of Ottawa, Ottawa, ON K1H 8 M5 Canada; University of Ottawa Center for Neuromuscular Disease, Ottawa, ON K1H 8 M5 Canada; College of Medicine & Veterinary Medicine, University of Edinburgh, Old Medical School, Teviot Place, Edinburgh, EH8 9XD UK

## Abstract

**Introduction:**

The term motor neuron disease encompasses a spectrum of disorders in which motor neurons are the lost. Importantly, while some motor neurons are lost early in disease and others remain intact at disease end-stage. This creates a valuable experimental paradigm to investigate the factors that regulate motor neuron vulnerability. Spinal muscular atrophy is a childhood motor neuron disease caused by mutations or deletions in the SMN1 gene. Here, we have performed transcriptional analysis on differentially vulnerable motor neurons from an intermediate mouse model of Spinal muscular atrophy at a presymptomatic time point.

**Results:**

We have characterised two differentially vulnerable populations, differing in the level neuromuscular junction loss. Transcriptional analysis on motor neuron cell bodies revealed that reduced Smn levels correlate with a reduction of transcripts associated with the ribosome, rRNA binding, ubiquitination and oxidative phosphorylation. Furthermore, P53 pathway activation precedes neuromuscular junction loss, suggesting that denervation may be a consequence, rather than a cause of motor neuron death in Spinal muscular atrophy. Finally, increased vulnerability correlates with a decrease in the positive regulation of DNA repair.

**Conclusions:**

This study identifies pathways related to the function of Smn and associated with differential motor unit vulnerability, thus presenting a number of exciting targets for future therapeutic development.

**Electronic supplementary material:**

The online version of this article (doi:10.1186/s40478-015-0231-1) contains supplementary material, which is available to authorized users.

## Introduction

Motor neuron diseases (MNDs) are a heterogeneous group of neurodegenerative disorders that are caused by a diverse array of factors, including both genetic and sporadic. The clinical severity can also vary widely, but MNDs are frequently very severe, causing fatality within months to years of diagnosis. Despite a range of causes and severities, motor neuron diseases are united by the common vulnerability of motor neurons. The reason why these cells are selectively vulnerable to the genetic or environmental insult is unknown. Importantly, however, not all motor neurons are equally affected [26]. There are some pools of motor neuron in which there are high levels of pathology throughout the motor unit, and high levels of motor neuron loss [6, 22, 27, 16]. In other pools in the same individual, there will be minimal evidence of motor unit pathology, even at late stages of disease. The reasons for this selective vulnerability are currently unknown, however this phenomenon creates an exciting and valuable opportunity to investigate the factors governing motor neuron vulnerability and pathology. By comparing different motor neuron groups we can investigate the molecular mechanisms underlying the disease, the molecular mechanisms underlying motor neuron pathology and the mechanisms that regulate motor neuron vulnerability.

Spinal muscular atrophy (SMA) is a childhood motor neuron disease caused by mutations and deletions within the survival motor neuron 1 gene (*SMN1*). There is a second partially functional copy of the *SMN* gene, termed *SMN2*. Due to a point mutation, *SMN2* predominantly produces a form of SMN that lacks exon 7, which is rapidly degraded. This gene therefore produces only low levels of the full length *SMN* transcript. The copy number of *SMN2* can vary, making it an important phenotypic modifier, with disease severity closely correlating to the number of copies of the *SMN2* gene. For this reason, SMA has a range of clinical severities, which are classified into types 0 to 4 based on age of onset and motor milestones achieved. Despite a degree of controversy in the field as to the cell types affected in SMA, it is clear that motor neurons are particularly vulnerable to reduced SMN levels. Indeed, SMA is typified by the loss of lower motor neurons and the atrophy of associated skeletal musculature.

In SMA patients there is evidence of pathology at the neuromuscular junction (NMJ) [37, 53]. Indeed in models of SMA, structural pathology in the motor neuron is first observed at the NMJ [40]. NMJs are lost early in the disease, with denervation evident in some muscles prior to symptom onset [40]. Additional defects at the NMJ include neurofilament accumulation, poor terminal arborisation, delayed post-synaptic maturation, defects in calcium handing and a disruption in synaptic vesicle release [10, 25, 28, 29, 34, 40, 44]. Importantly, not all NMJs are equally affected. This has been well characterised in mouse models of SMA, where there appears to be high levels of NMJ loss in some muscles and very low levels of NMJ loss in others [39, 40, 33]. This selective vulnerability has even been observed within single muscles, where there are areas of high levels of NMJ loss, and other areas showing no apparent denervation [40]. The reasons for this differential vulnerability are unclear. One study has investigated the various aspects of differentially vulnerable motor units, including motor unit size, muscle fibre type, NMJ size, branching patterns and Terminal Schwann cell number but found no correlation with differential vulnerability [49]. There is some evidence that differential vulnerability might correlate with a differential sprouting competence, presenting the possibility that relative plasticity can be an important modifier. Furthermore, recent work has shown that there are defects in synaptic remodelling at the NMJ in a mouse model of SMA [39]. However, this has not been shown to be a definitive modulator of vulnerability. The reasons for the variability in NMJ vulnerability are therefore currently unclear.

The reasons why motor neurons are selectively vulnerable to a reduction in Smn are currently undefined and the cellular functions for Smn have long been debated. The best described function for Smn is in pre-mRNA splicing [8, 57]. Growing evidence suggests that a reduction in Smn does not result in widespread splicing defects however it has been suggested that reduced Smn levels may cause splicing defects in a small subset of RNAs [36, 3]. Other roles for Smn have also been proposed, including transport of mRNAs [15] and a potential role as a translational regulator [45]. Ultimately, the precise cellular function of Smn that, when compromised in SMA, causes motor neuron pathology is unknown.

SMA research has been greatly facilitated by a number of mouse models [48]. These models are traditionally concentrated at the severe end of the spectrum, with a life expectancy of less than 14 days of age [31, 38, 23]. Despite their limitations, they have been instrumental in understanding the pathophysiology of SMA and in therapeutic development. The development of the *Smn*^*2B/-*^ mouse model of SMA has been an important addition to existing mouse models [5, 20]. This mouse was created by introducing a mutation in a splice enhancer site in the murine *Smn* gene, resulting in production of 10-15 % of normal Smn levels [20]. This mouse model displays all the hallmark pathologies associated with SMA but, with a phenotypic onset of around 10 days and a life span of around 28 days, has a slightly milder phenotype than most other models [5]. Importantly, unlike other mouse models, the *Smn*^*2B/-*^ mouse has a prolonged pre-symptomatic time period, thus allowing analysis of the events preceding motor neuron loss in SMA.

In this study, we have used the *Smn*^*2B/-*^ mouse model of SMA to investigate the transcriptional changes that occur in differentially vulnerable motor neurons. We have identified and defined muscles with differential levels of NMJ pathology and identified the time points associated with the onset of degeneration. At the pre-degenerative time points, we have used retrograde tracers to identify the motor neuron cell bodies which correspond to these differentially vulnerable NMJs and isolated them by laser capture micro-dissection. Vulnerable and less vulnerable motor neuron populations were isolated from both SMA and wild-type control mice. We then performed RNAseq on these differentially vulnerable motor neuron pools. Comparative bioinformatics analysis and functional clustering was performed to identify the transcriptional changes that correlate with reduced Smn levels, increased vulnerability and increased pathology. We demonstrate that reduced Smn levels are correlative with a decrease in transcripts implicated in ribosome, rRNA binding, ubiquitin and oxidative phosphorylation. We demonstrate a selective up regulation of cell death pathways in selectively vulnerable motor neurons, and demonstrate that an increase in these transcripts is observed prior to NMJ loss. Finally, we demonstrate that there is a decrease in markers of DNA repair in selectively vulnerable motor neurons. Overall this work details a four way comparative analysis of differentially vulnerable motor neurons using high resolution transcriptional profiling.

## Materials and methods

### Mouse maintenance

The *Smn*^*2B/-*^ mice [5] were established in our laboratory and maintained in the University of Ottawa vivarium on a C57BL/6 x CD1 hybrid background. Mice were sacrificed by exposure to rising CO_2_ levels or by cervical dislocation. All procedures were performed in accordance with institutional guidelines (Animal Care and Veterinary Services, University of Ottawa). *Smn*^*−/−*^*;SMN2* mice [23] were maintained in the animal facilities at the University of Edinburgh and were sacrificed by overdose of anaesthetic. Tissues were provided by generous agreement with Prof Tom Gillingwater. All procedures were carried out in accordance with the procedures approved and licenced by the Home Office, United Kingdom.

### Motor neuron labelling

Rhodamine conjugated dextran (RhDextran; 3000 MW; Molecular Probes) was administered under general anaesthesia [17]. For immunostaining, a fixable analogue of the RhDextran was used. Mothers were pre-dosed with buprenorphine prior to surgery. Mice were anesthetised by inhalation of isofluorane (2 % in 1:1 N_2_O/O_2_). For abdominal muscle labelling, a small incision between the last rib and xyphoid process was made and 5 μl of 5 % RhDextran was injected into the space between the external oblique muscle and the transversus abdominis muscle. For cranial muscle labelling, a small incision was made on the back of the neck, and 5 μl of RhDextran was injected into the space between the levator auris longus and auricularis superior muscles. Mice were allowed to recover from anaesthetic before being returned to standard cages. For muscle analysis, mice we sacrificed 24 h later and muscles were dissected, fixed in paraformaldehyde (PFA) and mounted on slides. For spinal cords and brainstems, mice were sacrificed 48 h later and spinal cords and brainstems were removed and snap frozen in liquid nitrogen in 50 % Tissue Tek O.C.T, 15 % sucrose in PBS.

### Laser capture microdissection of RhDextran labelled motor neurons

Spinal cords or brainstems were frozen and embedded as described above. Tissues were then sectioned using a cryostat at a thickness of 12 μm and mounted onto uncoated, uncharged glass slides and immediately frozen at −20 °C before being stored at −80 °C. Fluorescent motor neurons were then identified and imaged on an inverted epifluorescent microscope. During this process, slides were kept frozen using a freezing aerosol. Images were assembled and montaged using Adobe Photoshop and printed onto overhead transparencies. In the interim, slides were nissl stained and dehydrated as follows: 75 % EtOH, 30 s; ddH2O, 30 s; 1 % Toludine blue, 60 s; ddH2O, 30 s; 75 % EtOH, 30 s; 95 % EtOH, 30 s; 100 % EtOH x 3, 60 s; Xylene, 5 min; Air dry, 5 min. Steps were taken throughout to minimise RNAse exposure and DEPC water was used throughout.

LCM was performed using an Arcturus XT laser capture microscope from Applied Biosystems. Labelled motor neurons were identified by realigning the printed images of the fluorescent motor neurons with the nissl stained motor neurons on the LCM computer screen. This step was necessary due to the water soluble nature of the RhDextran, which is lost during LCM processing. Laser settings were optimised for motor neuron cell body size. Captured cells were snap frozen in qiazol (Qiagen) and stored at −80 °C until extraction of RNA.

### RNAseq

RNA was extracted using a Qiagen microRNA micro RNeasy kit as per manufacturer’s instructions. RNA was amplified using the ovation RNAseq system version 2 from Nugen according to manufacturer instructions and template DNA library construction was performed with the Encore Rapid Library System (Nugen). Four cycles of PCR were performed using reagents from the Ovation Ultralow kit (Nugen). After that, 36 cycles of single-end sequencing was performed with the Genome Analyzer IIx (Illumina). Reads were mapped to the mouse mm9 assembly using tophat (v1.4.0) using the transcripts from Ensembl release 67 to guide mapping. Quality control was performed using RNAseQC and FastQC. Relative transcript levels were compared using CuffDiff software v1.3 using the UCSC transcript model. Significance was considered with a Q-value of less that 0.05. The statistic package R was used to identify transcriptional changes which were common in more than one data set i.e. identifying transcriptional changes occurring in SMAv and SMAr motor neurons compared to their respective wild-types. Fold changes were converted to log2. Therefore no change, which is equivalent to a one fold change is equal to log_2_1, which equals 0. Therefore all numbers greater than 0 imply an up-regulation. All numbers less that 0 imply a down-regulation. As the biological consequences for a specific magnitude of change are not known, we have not applied any fold change restrictions to the data.

Functional clustering was performed using the functional annotation clustering tool available on the Database for Annotation, Visualization and Integrated Discovery (DAVID) online software from the National Institute of Allergy and Infectious Diseases [13]. Using this tool, enrichment analysis was performed using over 40 annotation categories. An algorithm was then applied which clustered the functional annotations based upon the degree of co-associated genes. Only those functional annotations that had a significant non-adjusted P value, were clustered. Those clusters with an enrichment score of >1.3 are considered significant. The enrichment score is a Log10 representation of the non-adjusted P value. Therefore an enrichment score of 1.3 is reflective of a P value of 0.05. This software also provides Kyoto Encyclopaedia of Genes and Genomes (KEGG) pathway annotation to identify specific cellular pathways that contain an enrichment for differentially expressed genes.

### qPCR

For q-RT-PCR on laser captured motor neurons, motor neuron cell bodies were isolated and RNA was extracted and amplified as above and cDNA was diluted to approximately 100 ng/μl. Transcripts were selected for validation based on having a high relative expression (based upon the read counts from the RNAseq results, preferably a read value of >50) balanced with a large change in expression level (ideally >1.5 fold). For whole spinal cord, RNA was extracted using a micro RNeasy kit (Qiagen) and 1 ug of RNA was used to perform reverse transcription using the RT^2^ First Strand kit (Qiagen). SYBR gene based Q-RT-PCR was performed using pre-optimised primers purchased from BioRad. Amplification was performed using KAPA SYBR fast universal PCR mastermix as per manufacturer instructions on a BioRad CFX connect real-time PCR detection system. Relative gene expression was calculated using the 2^-ΔΔcT^ formula [35]. β-actin and Y-Whaz were used as reference genes.

### Immuno-staining

For NMJ labelling, muscles were immediately dissected from recently sacrificed mice and fixed in 4 % PFA (Electron Microscopy Science) in PBS for 15 min. Post-synaptic AChRs were labelled with α-bungarotoxin (BTX) for 30 min. Muscles were permeabilised in 2 % Triton X-100 in PBS for 30 min, then blocked in 4 % bovine serum albumin (BSA)/1 % Triton X-100 in PBS for 30 min before incubation overnight in primary antibodies [Neurofilament (NF; 2H3) - Developmental Studies Hybridoma Bank; synaptic vesicle protein 2 (SV2) - Developmental Studies Hybridoma Bank; S100 – Dako; all 1:250] and visualised with Cy3-conjugated secondary antibodies [Cy3 goat anti-mouse; 1:250, Jackson]. Muscles were then whole-mounted in Dako Fluorescent mounting media. Images were taken with a Zeiss LSM-510 confocal microscope.

For spinal cord sections, spinal cords were removed from recently sacrificed mice and fixed overnight in 4 % PFA. Tissues were equilibrated in 30 % sucrose, embedded in 50 % Tissue Tek O.C.T, 15 % sucrose in PBS and sectioned at 12 μm on a cryostat. Sections were washed in PBS, permeabilised in 0.1 % Triton X-100 for 10 min and blocked in 4 % BSA for 30 min before exposure to primary antibodies (rabbit anti-pH2AX, Cell Signalling Technology; mouse anti-neurofilament heavy chain [SMI-32] BioLegend) overnight. Secondary antibodies (AlexaFluor 555 Goat anti-Rabbit, AlexaFluor 488 Rabbit anti-Mouse, both Life Technologies) were applied for a period of 2–4 h at a dilution of 1:200. Sections were counterstained in DAPI (Life Technologies) and NeuroTrace® 500/525 (Life Technologies) as per manufacturer instructions and mounted in mowoil® (Sigma Aldrich). Sections were imaged on a Zeiss LSM-510 confocal microscope. All laser settings were kept constant between images.

### Quantification and statistics

The percentage of fully occupied endplates was determined by classifying each endplate in a given field of view either fully occupied (pre-synaptic terminal (SV2 and NF) completely overlies endplate (BTX)), partially occupied (pre-synaptic terminal only partially covers endplate (BTX)), or vacant (no pre-synaptic label overlies endplate). At least 4 fields of view were analysed per muscle totalling >100 endplates per muscle.

For quantification of the number of labelled motor neurons following RhDextran injection, longitudinal sections of the anterior horn of the thoracic spinal cord or pons and medulla of the brainstem were cut and mounted sequentially on glass slides. The number of fluorescent motor neurons that were visible was counted per section. Quantification was done visually using a Zeiss Axiovert 200 M microscope.

pH2AX quantification was done using Image-J software. The average background intensity was subtracted from each image, leaving only the pixels with an intensity above background levels. The number of pH2AX foci per neuron was then quantified. All quantification was done with the experimenter blind to the experimental group.

All data was assembled and analysed using Microsoft Excel and GraphPad Prism. All figures were assembled using Adobe Photoshop.

## Results and discussion

### Differential motor unit vulnerability in the Smn^2B/-^ mouse model of SMA

**Fig. 1:**
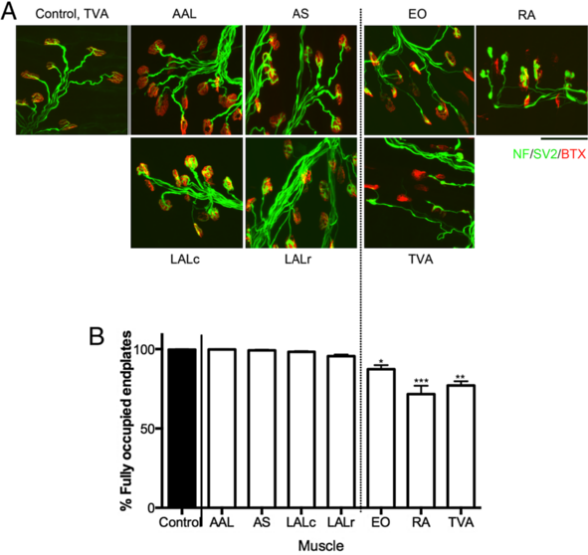
Neuromuscular junction analysis reveals differential vulnerability between cranial and abdominal muscles. **a** Confocal micrographs show neuromuscular junctions labelled with bungarotoxin (BTX, red) and antibodies against neurofilament and synaptic vesicle protein 2 (NF/SV2, green). Images are P21 *Smn* ^*2B/-*^ muscles (Adductor auris longus [AAL]; Auricularis Superior [AS]; Levator Auris Longus caudal and rostral bands [LALc, LALr]; External Oblique [EO]; Transversus Abdominis [TVA] and Rectus Abdominis [RA]. Control muscle is from P21 *Smn* ^*2B/+*^ (control) TVA. Note increase in denervated endplates and pre-synaptic swelling and poor pre-synaptic elaboration and endplate maturation in abdominal (EO, TVA, RA) muscles compared to cranial (LAL, AS, AAL) and control muscles. Scale bar = 75 μm. **b** Bar chart (mean ± SEM) showing percentage of fully occupied endplates in cranial and abdominal muscles in *Smn* ^*2B/-*^ mice at P21. ***P < 0.001, ** *P* < 0.01, *P < 0.05 by Kruskal-Wallis test with Dunn’s Multiple Comparison Test in comparison to control (*Smn* ^*2B/+*^ TVA)

Here we have used the *Smn*^*2B/-*^ mouse to investigate the transcriptional differences between differentially vulnerable motor units in SMA. To do this, we first identified two differentially vulnerable and experimentally accessible pools of motor neurons. We aimed to identify one pool of motor neurons in which we saw a high degree of NMJ loss, and another pool of motor neurons in which there was minimal or no evidence of NMJ loss at end stage of disease. For this study, it was important to us to use muscles that fulfilled the following criteria. Firstly, we aimed to use thin and flat muscles, which are easy to perform immunofluorescence on, thus allowing comprehensive analysis of the whole muscle. This was important so we could accurately quantify the level of morphological pathology present, and to ensure uniformity of pathology throughout the muscle. Secondly, muscles had to be experimentally accessible. This was important so tracer injections could be performed to specifically target the muscle group of interest with minimal trauma or risk of injection side effects, which may occur in the diaphragm or intercostal for example. Lastly, we felt it was preferable to use local groups of muscles, rather than single muscles. Clearly there is significant molecular variability between muscles. By using muscle groups, we minimised the contribution of inter-muscular variability. After investigating NMJ pathology in a range of muscles situated throughout the body, we defined our vulnerable group as those motor neurons innervating a group of abdominal muscles, specifically transversus abdominis (TVA), rectus abdominis (RA) and external oblique (EO). Collectively 20.5 % of NMJs were denervated by P21 in these muscles, and all these muscles display significant NMJ defects (Fig. 1). We observed defects such as neurofilament accumulation and poor terminal arborisation. Endplates appeared immature and there was a high number of vacant denervated endplates. Importantly, pathology was uniform throughout each muscle (data not shown). For our less vulnerable group of motor neuron, we chose to use motor neurons innervating a group of cranial muscles, encompassing levator auris longus (LAL), auricularis superior (AS) and adductor auris longus (AAL). Collectively, 97.9 % of NMJs were fully occupied at P21 in these muscles (Fig. 1b). Pre-synaptic terminals appeared more elaborate compared to those in abdominal muscles, and endplates displayed more complexity consistent with a more mature phenotype. Again, pathology was generally consistent throughout each muscle. One exception to this was a slight increase in NMJ pathology in the rostral band of the LAL muscle compared to the caudal band, where we observed 95.6 % of denervated endplates compared to 98.3 % in the LALc. Intriguingly, this is actually the opposite to what is observed in more severe mouse models of SMA [40]. Importantly, however, this denervation was still comparatively mild compared to abdominal muscles.

**Fig. 2:**
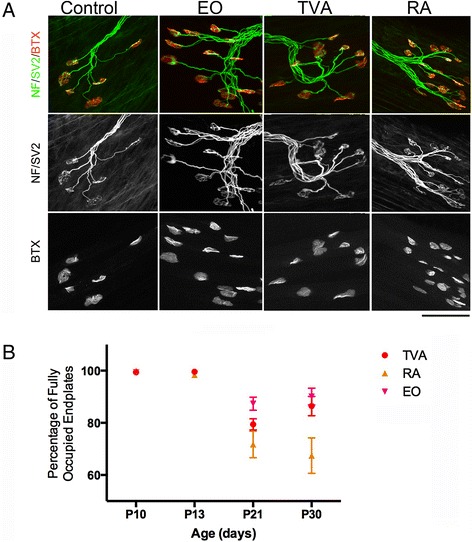
Time course analysis of abdominal NMJs reveals no denervation at pre-symptomatic stage (P10). **a** Confocal images show NMJs from P10 *Smn* ^*2B/-*^ abdominal muscles (External Oblique [EO]; Transversus Abdominis [TVA] and Rectus Abdominis [RA]) labeled with bungarotoxin (BTX, red) and antibodies against neurofilament and synaptic vesicle protein 2 (NF/SV2, green). Control is from *Smn* ^*2B/+*^ P10 EO muscle. Note that although there is evidence of some pre-synaptic swelling, there is no evidence of denervation. P10 is therefore defined as a pre-degenerative time point. Scale bar = 100 μm. **b** Scatter plot (mean ± SEM) shows the percentage of fully occupied endplates at P10, P13, P21 and P30 in individual TVA (red), RA (orange) and EO (magenta) muscles. Note that no degeneration is observed at P10. Loss of NMJs is progressive from P13 onwards. *N* = 4 per time point per muscle

It is important to note that our goal was to investigate the transcriptional differences between differentially vulnerable motor neurons prior to the onset of degeneration. We therefore aimed to identify a time point just prior to the onset of NMJ loss in vulnerable muscles. We performed a time course analysis of NMJ loss in the TVA, RA, IO and EO muscles. This revealed that the latest time point at which there was no evidence of NMJ loss was P10 (Fig. 2). Analysis of NMJs at P10 revealed no denervation in any of the abdominal muscles analysed (Fig. 2a). We have therefore defined this as a pre-degenerative time point and aimed to use this time point for all subsequent transcriptional analysis.

### Motor unit tracing

To identify the motor neuron cell bodies that correspond to the differentially vulnerable pools of NMJs, we used rhodamine-conjugated dextran as a retrograde tracer. For abdominal muscle, RhDextran was injected into the space between the TVA and RA/EO muscles. For cranial muscles, RhDextran was injected into the space between the LAL and AS/AAL muscles. For initial experiments, mice were sacrificed 24 h later and muscles were analysed for the presence of RhDextran. Analysis of abdominal muscles revealed strong staining in the superior parts of the EO, RA and TVA (data not shown). Importantly, the dye remained relatively local to the site of injection, and did not leach to surrounding intrinsic back or appendicular muscles. Equivalent analysis was performed in the cranial muscles. Strong staining was observed in the LAL, AS and AAL muscles and again dye was observed to remain local to the site of injection, with minimal leakage to surrounding muscles or to the contralateral side. This analysis confirmed that we were labeling the targeted muscles specifically and robustly.

**Fig. 3:**
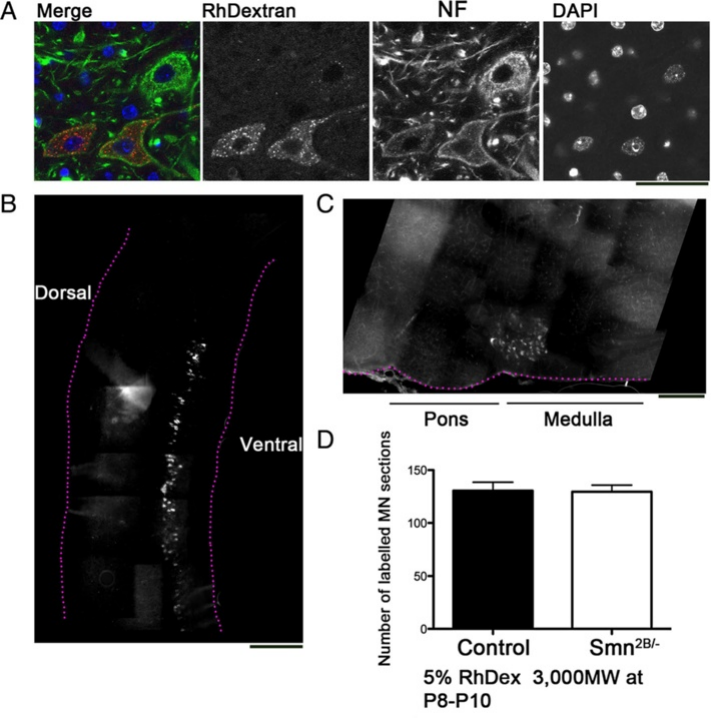
RhDextran can be used to label cell bodies corresponding to differentially vulnerable muscles. **a** Confocal images show motor neuron cell bodies labeled with Neurofilament (NF, green) and DAPI (blue) following intramuscular injection of RhDextran (red). Note positive staining in 2 of the 3 motor neurons present. Scale bar = 25 μm. **b**, **c** Montaged fluorescent micrographs show RhDextran labeling in the spinal cord and brainstem following injection of RhDextran into the abdominal or cranial muscle groups, respectively. Scale bar = 300 μm (**b**), 500 μm (**c**). **d** Bar chart (Mean ± SEM) showing number of labeled motor neurons in the spinal cord 48 h after abdominal muscle injection of 5 % RhDextran into P8 control (wild-type) or *Smn* ^*2B/-*^ mice. *N* = 3 mice per genotype

For subsequent experiments, mice were sacrificed 48 h following RhDextran injection and spinal cords and brainstems were analysed for the presence of RhDextran staining. This revealed a discrete cluster of motor neurons in the thoracic spinal cord, which correspond to the motor neurons innervating the abdominal muscles, and a cluster of labeled motor neurons in the facial nucleus of the brainstem, corresponding to those motor neurons innervating the cranial muscles (Fig. 3). Quantification of the number of labeled motor neurons between *Smn*^*2B/-*^ mice and wild-type control animals revealed no difference between groups (Fig. 3d). This suggests that labeling efficiency is not compromised in *Smn*^*2B/-*^ mice. This was important to consider given previous work reporting defects in axon transport in SMA mouse models [11].

### Transcriptional analysis of differentially vulnerable motor neurons

**Fig. 4:**
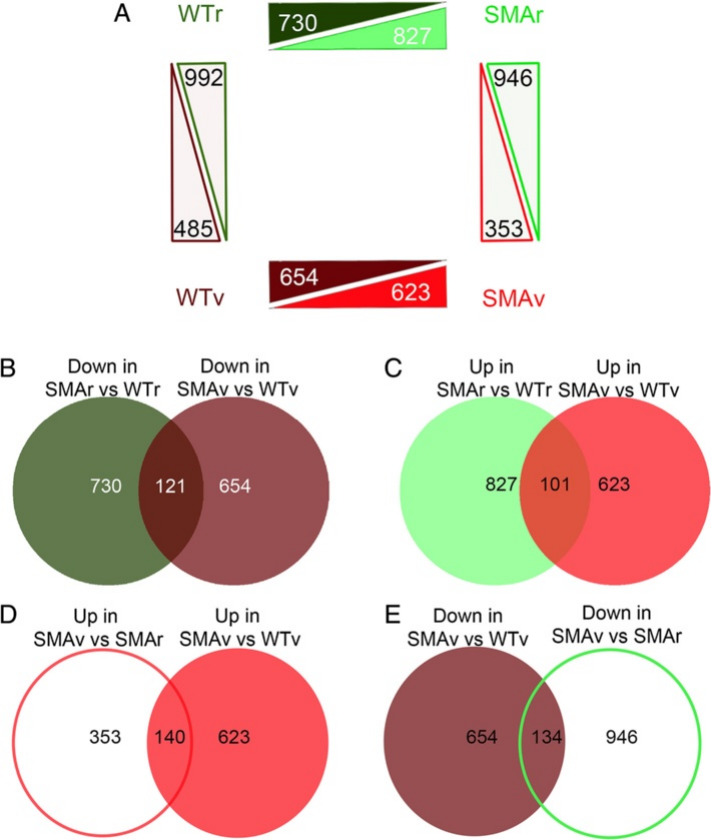
RNAseq results show large number of differentially regulated transcripts. **a** Schematic diagram shows number of statistically significant altered transcripts that were up or down regulated between the four experimental groups (WTr and SMAr, less vulnerable [cranial] motor neurons from wild-type and *Smn* ^*2B/-*^ mice respectively; WTv and SMAv, vulnerable [abdominal/thoracic] motor neurons from wild-type and *Smn* ^*2B/*-^ mice respectively). **b**-**e** Venn diagrams showing the number of common transcriptional changes between each of the primary comparisons which were made in panel **a**. Diagrams show number of changes which went down (**b**) or up (**c**) in both SMAv and SMAr motor neurons compared to their respective wild-types, and number of changes which went up (**d**) or down (**e**) in SMAv motor neurons compared to both SMAr and WTv motor neurons

Following identification of suitable differentially vulnerable motor neuron populations, and validation of the RhDextran labeling methodology, laser capture micro-dissection (LCM) was used to isolate the cell bodies corresponding to these neurons. The motor neuron cell bodies were isolated from *Smn*^*2B/-*^ and wild-type mice, with approximately 250 motor neurons from 2–3 mice per biological replicate. This resulted in four experimental groups. Vulnerable (abdominal) motor neurons from *Smn*^*2B/-*^ and wild-type mice (SMAv and WTv respectively) and less vulnerable (cranial) motor neurons from *Smn*^*2B/-*^ and wild-type mice (SMAr and WTr), respectively. Transcriptional analysis was performed using RNAseq and expression profiles were compared between each experimental group (Additional file 1). For each comparison, around 17,500 genes were identified and compared. From this number, there were around 1200 to 1500 transcriptional changes between each of our four comparative groups (Fig. 4). Encouragingly, preliminary analysis of the data revealed a number of changes that have been previously implicated in pathology in SMA or in motor neuron vulnerability. For example, we observed a significant 4.8 Log_2_ fold up regulation of cyclin dependant kinase inhibitor 1A (CDKN1a) between SMAv and WTv motor neurons, and a 2.8 Log_2_ fold up regulation of CDKN1a between SMAr and WTr motor neurons. Interestingly, a significant up-regulation of CDKN1a has been reported in numerous other models of SMA, including in other mouse models of SMA [60] and embryonic stem cell derived motor neurons from SMA mouse models [58]. We revealed a significant 1.3 Log_2_ fold down-regulation of chondrolectin (Chodl), which has previously been described in other mouse models of SMA [3, 60]. There was a significant 1.03 fold Log_2_ up-regulation of the Fused in Sarcoma transcript (Fus), which has been implicated in motor neuron pathology in amyotrophic lateral sclerosis (ALS), and the protein has been shown to interact with Smn [18, 59]. We also observed a significant 3.5 Log_2_ fold down regulation of insulin like growth factor 1 (IGF1) which has previously been reported in serum and liver of a SMA mouse model [24]. Interestingly, we did not observe any change in Fus, IGF1 or Chodl in SMAr motor neurons compared to WTr. As these genes have also been implicated in motor neuron pathology in amyotrophic lateral sclerosis [9, 30, 52, 56], this perhaps indicates that these genes are involved in motor neuron pathology, which occurs as a secondary effect to the loss of Smn protein. These genes are therefore potential common downstream regulators of neuronal pathology in a variety of different motor neuron diseases.

### What transcriptional changes occur in motor neurons when Smn is absent: transcriptional differences in vulnerable motor neurons between SMA and WT mice

**Fig. 5:**
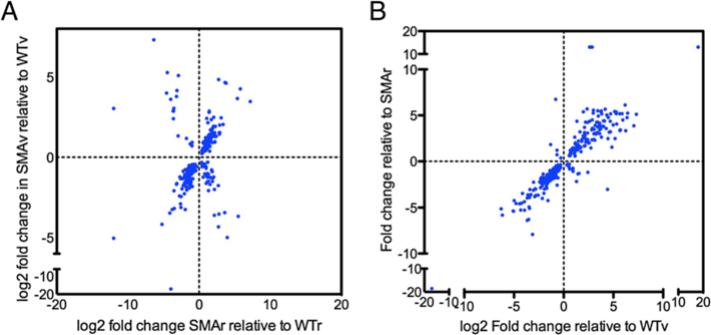
The majority of common transcriptional changes occur with the same directional regulation. **a** Scatter plots show correlation in fold change in transcripts that were statistically significantly altered between both SMAv and SMAr motor neurons in comparison to their wild-type equivalent. Note that most (222 of the 273) common changes identified shared the same directional regulation. **b** Scatter plots show correlation in fold change in transcripts that were statistically significantly altered in SMAv motor neurons compared to both SMAr and WTv. Note that 274 of the 292 common changes identified shared the same directional regulation

We first aimed to use this data to investigate the cellular processes disrupted when Smn levels are decreased. We reasoned that Smn levels are reduced in both SMAv and SMAr motor neurons compared to their respective wild-type counterparts. We therefore looked for the transcriptional changes that occurred in both of these populations. In SMAr motor neurons, there were 1557 transcripts, which were statistically up or down regulated compared to WTr motor neurons. In SMAv motor neurons, there were 1277 transcripts, which were statistically up or down regulated compared to WTv motor neurons. Of these changes, 273 were found in both comparisons and the majority (222) showed a common directional change, with 121 being up-regulated and 101 being down regulated (Fig. 4, Fig. 5a, Additional file 2).

**Fig. 6:**
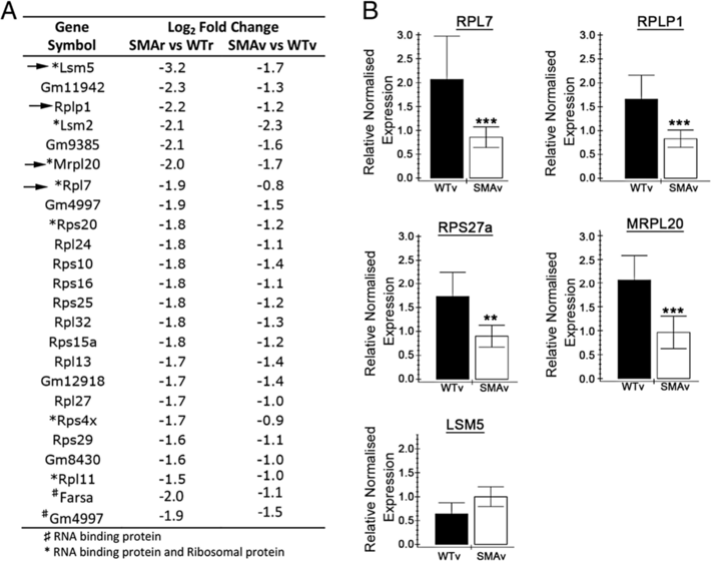
Down-regulation of transcripts involved in the ribosome and rRNA binding. **a** Gene list show transcripts associated with the ribosome or rRNA binding which were statistically altered in SMAv and SMAr motor neurons compared to their respective wildtypes. No annotation implies just associated with the ribosome; *rRNA binding proteins and ribosome; ♯rRNA binding proteins not implicated in the ribosome. Arrow indicates transcripts that were investigated by qPCR. **b** Bar charts (Mean ± SEM) showing relative expression of RPL7, RPLP1, RPS27a, MRPL20 and LSM5 in WTv and SMAv motor neurons. *N* = 4 biological replicates, each approximately 200 motor neurons from 2–3 mice. Note that all qPCR results confirmed RNAseq results with the exception of LSM5 for which there was no change. ***P < 0.001, **P < 0.01 by Mann Whitney *U* test

Functional clustering of these transcripts was performed using DAVID bioinformatics resources version 6.7. This revealed a number of significantly altered functional clusters (Table 1). As we were looking for transcripts implicated in the function of Smn, we decided to focus on transcripts that are down-regulated in SMAv and SMAr motor neurons. The most significant clusters which were down-regulated comprised of transcripts involved in the ribosome and cytosolic ribosome. This was closely followed by transcripts implicated in rRNA binding. Indeed there was considerable overlap between these three functional clusters, with the majority of rRNA binding proteins also implicated in the ribosome (Fig. 6). In order to confirm this down regulation of transcripts involved in rRNA binding and ribosome, we performed qPCR validation on new biological replicates of cDNA samples prepared from motor neurons isolated as described above, and compared transcript levels between SMAv and WTv samples. This confirmed a down-regulation of RPL7, RPLP1, RPS27a and MRPL20 (Fig. 6b). The only transcript that did not validate was LSM5.

**Table 1:** Statistically altered functional clusters of transcripts that are differentially expressed in SMAv and SMAr compared to their respective WTs

Down in SMA		Up in SMA	
Cluster	Enrichment	Cluster	Enrichment
Ribosome/Translation	21.7	Repeat 2-2	2.4
Cytosolic Ribosome	4.7	RNA binding/Splicesosome	2.2
rRNA binding	4.4	Heat-shock	1.7
Ubiquitin	2.1		
Oxidative Phosphorylation	1.6		

*Listed clusters are generated from functional annotations which were significantly enriched during functional annotation clustering. Significant enrichment was considered to be those clusters with an enrichment score of >1.3*

The data presented above therefore indicated that there is a down regulation of factors involved in the ribosome, particularly in those involved in rRNA binding. Smn has long been established as an RNA binding protein, with work generally focused on its role as an mRNA binding protein, and its role on pre-mRNA splicing and mRNA transport. Less is known about its potential role in rRNA metabolism. It is easy to speculate that reduced Smn levels may disrupt the production or assembly for rRNA and lead to downstream defects in ribosomal function. Furthermore, Smn has recently been identified as a negative regulator of translation [45]. Smn has also been shown to localise to the nucleolus and interact with non-ribosomal nucleolar proteins, suggesting that it has a role in ribonucleoprotein complex assembly [32, 54]. Interestingly, recent work has identified that mutations in genes involved in ribosome subunit assembly and maturation lead to motor neuron specific defects in Spinal Muscular Atrophy with Respiratory Distress (SMARD) [7, 12, 19]. This work therefore creates an intriguing parallel between SMA and SMARD and warrants further work into ribosome assembly and function in Smn depleted motor neurons.

**Fig. 7:**
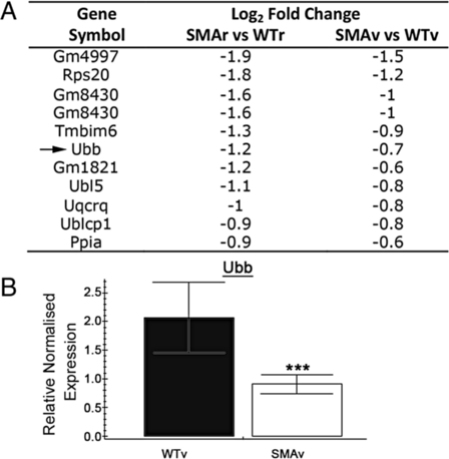
Down-regulation of transcripts involved in Ubiquitination. **a** Gene list show transcripts associated with ubiquitin which were statistically significantly altered in SMAv and SMAr motor neurons compared to their respective wildtypes. Arrow indicates transcript that was investigated by qPCR. **b** Bar chart (Mean ± SEM) showing relative expression of Ubiquitin (Ubb) in WTv and SMAv motor neurons. *N* = 4 biological replicates, each approximately 200 motor neurons from 2–3 mice. Note that qPCR results confirmed down-regulation of Ubb. ***P < 0.001 by Mann Whitney *U* test

The functional clustering also revealed a down regulation of transcripts involved in ubiquitin metabolism (Table 1, Fig. 7). The down regulation of ubiquitin (Ubb) was confirmed by qPCR (Fig. 7b). A disruption in ubiquitin homeostasis has recently been reported in SMA mouse models. Specifically, the authors observed a disruption in proteins implicated in ubiquitination in proteomic analysis on synapses from P1 SMA mice and a marked reduction in ubiquitin activating enzyme 1 (UBA1) levels in spinal cord and muscle from SMA mice [55]. They suggest this down-regulation in UBA1 leads to an increase in beta-catenin levels, and inhibition of beta-catenin signalling rescued motor neuron specific defects in zebrafish and mouse models of SMA. This work therefore confirms the disruption of this pathway and promotes further work to dissect the mechanism by which a reduction in Smn levels lead to a disruption in ubiquitin homeostasis.

**Fig. 8:**
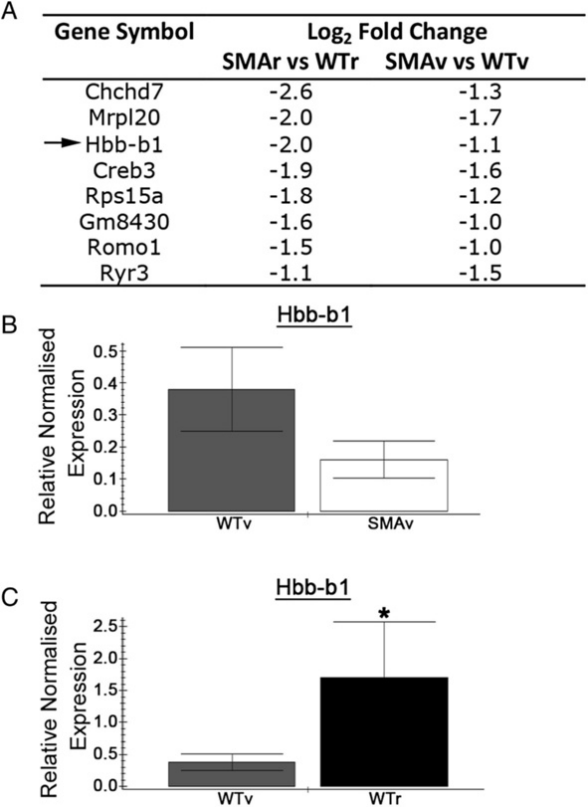
Down-regulation of transcripts involved in oxidative phosphorylation. **a** Gene list show transcripts associated with oxidative phosphorylation which were statistically altered in SMAv and SMAr motor neurons compared to their respective wildtypes. Arrow indicates transcript that was investigated by qPCR. **b**, **c** Bar charts (Mean ± SEM) showing relative expression of haemoglobin beta chain (Hbb-b1) in WTv and SMAv motor neurons (**b**) and in WTv compared to WTr motor neurons. Note that qPCR results confirmed down-regulation of Hbb-b1 in SMAv motor neurons and also revealed an increase in Hbb-b1 levels in less vulnerable (WTr) motor neurons compared to vulnerable motor neuron (WTv). *N* = 4 biological replicates, each approximately 200 motor neurons from 2–3 mice. *P < 0.05 by Mann Whitney *U* test

We also observed enrichment for transcripts involved in oxidative phosphorylation (Table 1, Fig. 8). Encouragingly, oxidative phosphorylation was also the top hit on a proteomic screen on synapses from a P1 SMA mouse model [55]. Of particular note, we reveal a down regulation of haemoglobin beta 1 (Hbb-b1) which was confirmed by qPCR between SMAv and WTv motor neurons (Fig. 8b). Interestingly, we also noted a strong increase in Hbb-b1 levels in WTr motor neurons compared to WTv. This suggests that Hbb levels may be higher in less vulnerable motor neurons. This makes haemoglobin an exciting candidate as a regulator of motor neuron vulnerability. Indeed, recent work has demonstrated that haemoglobin subunits are expressed in neurons in both rats and humans [41, 43] and colocalised with the mitochondria inner membrane [47]. They have been observed to be up-regulated in response to ischemic-reperfusion injury in rats and in an *in vitro* ischemic injury model in primary cortical neuron cultures [21]. Furthermore, haemoglobin chains have been shown to be down-regulated in a rat model of chronic stress [1], in microglia, astrocytes and brain mitochondria from aged mice [42, 47]. The role of haemoglobin in neurons is unclear, although it been suggested to act as an oxygen carrier or to be protective against oxidative stress [2, 46]. We may speculate about the relationship between Smn and haemoglobin, however it is perhaps more likely that the down-regulation of Hbb transcripts that we observed is a downstream consequence of the cellular defects caused by a reduction in Smn levels. However, as outlined above, there is strong evidence that haemoglobin can influence pathways implicated in neuronal vulnerability, and this makes it an exciting candidate for further investigation.

### Up-regulation of transcripts involved in programmed cell death in selectively vulnerable motor neurons that precedes NMJ pathology

**Fig. 9:**
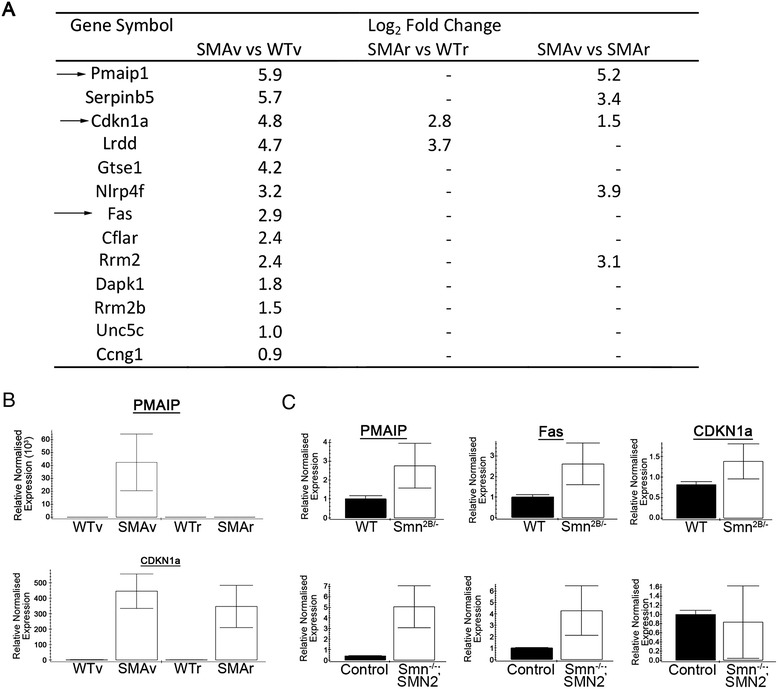
Selective up-regulation of transcripts involved in cell death in vulnerable motor neurons. **a** Gene list show transcripts associated with cell death which were statistically significantly altered in SMAv compared to WTv motor neurons. Arrow indicates transcripts that were investigated by qPCR. **b** Bar charts (Mean ± SEM) showing relative expression of PMAIP and CDKN1a in WTv, SMAv, WTr and SMAr motor neurons. Note that qPCR results confirmed up-regulation of PMAIP and CDKN1a in SMAv motor neurons compared to WTv. There was also a significant up-regulation of CDKN1a in SMAr motor neurons. *N* = 4 biological replicates, each approximately 200 motor neurons from 2–3 mice. **c** Bar charts (Mean ± SEM) showing relative expression of PMAIP, Fas and CDKN1a in whole spinal cord from *Smn* ^*2B/-*^ and *Smn* ^*−/−*^ *;SMN2* mice compared to wild-type or *Smn* ^*+/+*^ *;SMN2* (control) respectively. This showed an up-regulation of all transcripts in SMA mouse models, with the exception of CDKN1a in *Smn* ^*−/−*^ *;SMN2* samples. Note that this demonstrates the up-regulation of transcripts associated with cell death pathways can be seen at pre-symptomatic time points in two different mouse models of SMA. ***P < 0.001, *P < 0.05 by Mann Whitney *U* test

Functional clustering of the transcriptional changes occurring between SMAv and WTv motor neurons revealed a number of clusters, most of which were common with the functional clustering of changes between SMAr and WTr motor neurons (Table 2). One functional cluster that was only observed in SMAv *vs.* WTv motor neurons, was an up-regulation of factors involved in cell death (Fig. 9). This functional cluster was not significantly altered in equivalent analysis on SMAr *vs.* WTr motor neurons. There were 13 transcripts that are associated with cell death pathways, which were up-regulated in SMAv motor neurons compared to SMAr (Fig. 9a). Of these, only 2 were statistically up-regulated in SMAr motor neuron compared to WTr (Fig. 9a). Of particular note, 5 of these transcripts were up-regulated in SMAv motor neurons compared to SMAr (Fig. 9a). KEGG pathway analysis revealed that a number of these up-regulated transcripts pertain to the P53 signalling pathway. Specifically, the RNAseq results indicated a 4.8 Log_2_ fold and 5.9 Log_2_ fold up-regulation of PMAIP and CDKN1a respectively. This up-regulation was confirmed by qPCR on cDNA from laser captured motor neurons. An up-regulation of Fas, PMAIP and CDKN1a was also seen in cDNA deriving from whole spinal cord from P10 *Smn*^*2B/-*^ mice (Fig. 9c). A similar up-regulation was observed in spinal cord from pre-symptomatic (P2) *Smn*^*−/−*^*;SMN2* mice, a more severe mouse model of SMA (Fig. 9c).

**Table 2:** Statistically altered functional clusters of transcripts that are differentially expressed in SMAv *vs.* WTv

Down in SMAv		Up in SMAv	
Cluster	Enrichment	Cluster	Enrichment
Ribsome	10.7	Metal Ion Binding	3.2
Mitochondrial Membrane	2.7	Nucleoplasm	3.2
Ubl Conjugation	2.3	RNA binding	2.6
Mitochondria	2.14	Transcriptional Regulation	2.5
Bone Development	1.6	Cytoskeleton	2.1
Mitochondrial Ribosome	1.55	mRNA processing	2.1
Focal Adhesion	1.52	Nuclear Speck	1.6
Mitochondrial Respiratory Chain	1.39	Chromatin Regulator	1.6
		Transcriptional Activator	1.6
		Cell death	1.3

*Listed clusters are generated from functional annotations which were significantly enriched during functional annotation clustering. Significant enrichment was considered to be those clusters with an enrichment score of >1.3*

The up-regulation of factors that are strongly implicated in apoptotic pathways may at first glance appear unsurprising. However, we must remember that this up-regulation is occurring in two different mouse models of SMA at a pre-degenerative time point, prior to any NMJ loss. This suggests that cell death pathways are activated at the cell body prior to pathology at the NMJ. A central debate in the SMA research field has been whether SMA is due to a loss of a central housekeeping function for Smn, or whether it is due to a loss of a specific axonal or synaptic role for Smn [8, 15]. The observation that NMJs are lost so early in the disease has often been used as evidence for the latter. The data presented here suggests that cell death pathways are activated before degenerative events can be observed at the axon or NMJ. Clearly, this work does not eliminate a role for Smn in the axon or synapse. It also remains possible that defects in this synaptic or axonal role leads to cell death activation at the cell body, which is followed by the withdrawal of synaptic and axonal compartments. However, it is intriguing to draw parallels to congenic myasthenia syndromes, which are caused by specific synaptic defects [14]. In these conditions there is profound synaptic dysfunction and denervation, however this does not lead to activation of cell death pathways and motor neuron cell body loss. Further work is clearly required to determine the time course and specific location of motor unit pathology in SMA and investigate whether synaptic defects in SMA are a cause or consequence of cell death pathway activation at the cell body.

### What transcriptional changes correlate with motor neuron vulnerability: Transcriptional differences selectively occurring in vulnerable motor neurons

In this study we were particularly keen to identify regulators of motor neuron vulnerability. For this analysis, we reasoned that any changes that occurred only in SMAv motor neurons compared to either WTv motor neurons, or to SMAr motor neurons, could be specifically implicated in NMJ and motor unit pathology. We therefore looked for transcriptional changes that occurred in SMAv motor neurons compared to SMAr and compared to WTv. By comparing SMAv and SMAr motor neurons, we identified 1299 transcripts that were up or down-regulated (Fig. 4). As highlighted above, when comparing SMAv and WTv motor neurons, we identified 1277 transcripts that were up or down-regulated (Fig. 4). Of the changes that were identified in these two comparisons, 292 were common, and notably 94 % occurred with the same directional regulation (Fig. 5b). Indeed we found 140 and 134 transcriptional changes which were up or down regulated respectively in SMAv motor neurons compared to both WTv and SMAr (Additional file 3). Of particular note, both IGF1 and IGF2 were significantly down-regulated in SMAv motor neurons. IGF1 has previously been reported to be down-regulated in SMA models, and increasing IGF1 levels have been shown to be phenotypically beneficial [4, 50, 51]. IGF2 levels have been associated with differential neuronal vulnerability in an ALS model, down-regulated in an experimental model of stress, and suggested to have neuroprotective qualities against excitotoxicity [1, 22]. RNAseq results also implied that PMAIP and CDKN1a were selectively up-regulated in SMAv motor neurons. For PMAIP, this result was confirmed by qPCR (Fig. 9).

**Fig. 10:**
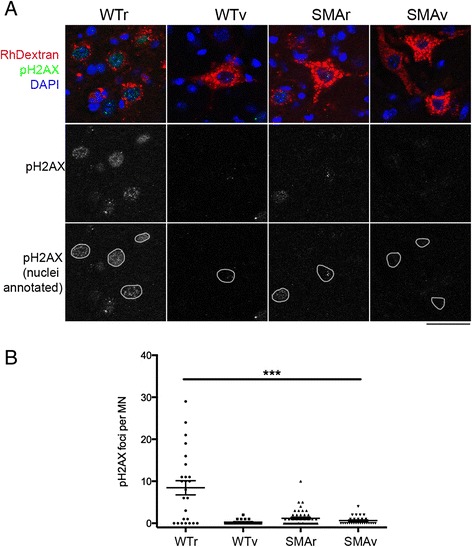
Increase in markers of DNA repair complexes correlate with decreased vulnerability. **a** Confocal images show motor neurons from WTr, WTv, SMAr and SMAv motor neuron labeled with RhDextran (red), DAPI (blue) and antibodies against phosphorylated version of H2A histone family member X (pH2AX, green), which is a marker of DNA repair complexes. Note that the highest levels of pH2AX staining were observed in WTr motor neurons. Scale bar = 50 μm. **b** Scatter plot quantification of the number of pH2AX positive foci per motor neuron in WTr, WTv, SMAr and SMAv motor neurons. There was a decrease in staining in WTv motor neurons, which suggests decreased DNA repair complexes correlate with increased vulnerability. There was also a decrease in DNA repair complexes in both SMAv and SMAr motor neurons. *N* = 4 spinal cord and 25/21/48/27 motor neurons per group, *** *P* <0.001 WTr compared to WTv, SMAr and SMAv

Functional clustering of these changes highlighted a number of cellular pathways, which are detailed in Table 3. Due to their enrichment selectively in vulnerable motor neurons, these clusters are all potentially of interest and worthy of further investigation. We were particularly interested to note the decrease in factors involved in the positive regulation of DNA repair. The transcripts within this functional cluster are listed in Table 4. This presents the possibility that the response to DNA damage is decreased in selectively vulnerable motor neurons. In order to investigate this further, we looked at phospho-histone H2AX (pH2AX) levels in differentially vulnerable motor neurons (Fig. 10). pH2AX is one of the first proteins to be recruited to sites of DNA damage, and recruits other proteins involved in DNA repair to form a DNA repair complex. Immunohistochemical staining of differentially vulnerable motor neurons with antibodies against pH2AX revealed a marked increase in foci number in WTr motor neurons compared to WTv. This suggests that there is a basal increase in the level of DNA repair occurring in less vulnerable motor neurons. The number of pH2AX foci was reduced in both SMAv and SMAr motor neurons compared to WTr, suggesting a further decrease in the levels of DNA repair in *Smn*^*2B/-*^ motor neurons. Interestingly, there remained a trend for increased DNA repair in SMAr motor neurons compared to SMAv. This data indicated that the number of DNA repair complexes correlates with relative vulnerability.

**Table 3:** Statistically altered functional clusters of transcripts that are differentially expressed in SMAv compared to WTv and SMAr

Down in SMAv		Up in SMAv	
Cluster	Enrichment	Cluster	Enrichment
Metal Ion Binding	3.07	Mitochondrial inner membrane	1.38
Positive regulation of transcription	2.52		
Membrane	1.73		
Chromatin/methylation	1.47		
Positive regulation of DNA repair	1.38		
Nucleotide Binding	1.35		

*Listed clusters are generated from functional annotations which were significantly enriched during functional annotation clustering. Significant enrichment was considered to be those clusters with an enrichment score of >1.3*

**Table 4:** Genes implicated in DNA repair that were down regulated in SMAv motor neurons compared to both SMAr and WTv

Gene symbol	Log_2_ fold change	
	SMAv *vs.* WTv	SMAv *vs.* SMAr
EYA1	−3.0	−3.1
C230052I12RIK	−2.2	−1.7
SREBF1	−1.8	−1.9
CHD3	−1.5	−1.8
FTSJD2	−1.2	−1.1
CHD8	−1.2	−1.1
KDM1B	−1.2	−1.6
BRE	−1.1	−1.2
HBB-B1	−1.1	−1.7
MLH3	−1.0	−1.3
MLL5	−0.9	−1.1
ARPP21	−0.6	−1.8

The data presented above provide preliminary evidence that an increase in DNA repair can be neuroprotective. It is unclear whether this is because there is a greater requirement for DNA repair in less vulnerable motor neurons, or whether the requirement is similar in both cell populations, but DNA repair is just more efficient in less vulnerable cells. The DNA damage repair system is a crucial system to maintain genomic integrity, which is especially relevant for terminally differentiated and long-lived cells such as neurons. It is easy to speculate how an increase in the activity in the basic cellular repair mechanisms could be neuroprotective. Indeed, DNA damage can be a key mechanism by which apoptosis is induced. The increase in DNA repair in less vulnerable motor neurons may reduce the likelihood of apoptotic activation. A number of hypotheses state that stressed cells cope better with additional stress. It is possible that this basal higher level of cellular stress primes selective motor neuron populations to cope better with the additional stresses when Smn levels are reduced. Clearly this idea requires further validation in this context, however this has important implications both for development of neuroprotective strategies, and for understanding the mechanisms by which sub-populations of neurons are rendered more vulnerable to a variety of insults.

## Conclusions

In summary, the data presented above represent detailed transcriptional analysis on differentially vulnerable motor neurons from an SMA mouse model at a pre-symptomatic time point. They highlight a number of pathways which are disrupted upon Smn depletion, including a reduction of transcripts involved with the ribosome, rRNA binding, ubiquitination and oxidative phosphorylation. Subsequent work is required to ascertain the mechanisms by which reduced Smn levels impact upon these pathways. We have also revealed an early and selective up-regulation of cell death pathways, and it will now be key to understand how cell death pathway activation relates to the time course of pathology within the motor unit. Finally, we show that, among a number of pathways of potential interest, an increase in DNA damage repair complexes correlate with a reduced vulnerability. Future efforts dissecting the mechanism of this protection are now warranted.

## Additional files

Additional file 1: Table S1. Table shows a summary of the sequencing data obtained from the RNAseq analysis, as analysed using Tophat software. Table show the number of reads (reads), the percentage which mapped to a unique location (% unique), the percentage which mapped to a distinct location (% distinct), the number of mapped location (mapped locations), the number of mapped reads (mapped reads) and the percentage of 10.1186/s40478-015-0231-1 reads which were mapped (% mapped) for each of the 8 samples. (DOCX 53 kb) Additional file 2: Table S2. Table show a list of genes which are differentially expressed between SMAr and SMAv motor neurons when compared to their respective wild-types. (DOCX 113 kb) Additional file 3: Table S3. Table show a list of genes which are differentially expressed between SMAv motor neurons when compared to SMAr or Wtv motor neurons. (DOCX 113 kb)
