# Supplementary material for: Transcriptional profiling of differentially vulnerable motor neurons at pre-symptomatic stage in the Smn2b/- mouse model of spinal muscular atrophy
Source: Acta Neuropathol Commun. 2015 Sep 15;3:55. doi: 10.1186/s40478-015-0231-1 (PMC4570693; doi:10.1186/s40478-015-0231-1)
Supplement: Additional file 1: Table S1. — Table shows a summary of the sequencing data obtained from the RNAseq analysis, as analysed using Tophat software. Table show the number of reads (reads), the percentage which mapped to a unique location (% unique), the percentage which mapped to a distinct location (% distinct), the number of mapped location (mapped locations), the number of mapped reads (mapped reads) and the percentage of 10.1186/s40478-015-0231-1 reads which were mapped (% mapped) for each of the 8 samples. (DOCX 53 kb) [file 40478_2015_231_MOESM1_ESM.docx]

**Supplementary Table 1: Summary of sequence data generated from RNAseq using Tophat software mapping to mouse mouse mm-9 assembly**

| **Sample** | **Reads** | **% unique** | **% distinct** | **Mapped Locations** | **Mapped Reads** | **% mapped** |
| --- | --- | --- | --- | --- | --- | --- |
| WTr-1 | 34,357,529 | 41.87% | 49.04% | 35,997,510 | 31,786,793 | 92.52% |
| WTr-2 | 30,999,478 | 33.75% | 41.37% | 33,262,549 | 28,449,542 | 91.77% |
| SMAr-1 | 41,748,401 | 30.16% | 35.87% | 43,528,428 | 38,283,253 | 91.70% |
| SMAr-1 | 21,470,905 | 30.39% | 35.85% | 22,914,228 | 19,621,061 | 91.38% |
| WTv-1 | 30,955,646 | 30.49% | 36.12% | 32,642,295 | 28,069,777 | 90.68% |
| WTv-2 | 37,346,743 | 30.87% | 37.72% | 39,710,943 | 34,628,676 | 92.72% |
| SMAv-1 | 32,994,286 | 30.33% | 36.52% | 34,826,958 | 29,566,950 | 89.61% |
| SMAv-2 | 32,525,361 | 32.46% | 38.87% | 36,360,051 | 29,445,476 | 90.53% |
