# Supplementary material for: Transcriptional profiling of differentially vulnerable motor neurons at pre-symptomatic stage in the Smn2b/- mouse model of spinal muscular atrophy
Source: Acta Neuropathol Commun. 2015 Sep 15;3:55. doi: 10.1186/s40478-015-0231-1 (PMC4570693; doi:10.1186/s40478-015-0231-1)
Supplement: Additional file 2: Table S2. — Table show a list of genes which are differentially expressed between SMAr and SMAv motor neurons when compared to their respective wild-types. (DOCX 113 kb) [file 40478_2015_231_MOESM2_ESM.docx]

**Supplementary Table 2: Statistically altered transcriptional changes in both SMAr and SMAv motor neurons compared to their respective wild-types**

| **Official Gene Symbol** | **SMAr vs WTr** | **Q Value** | **SMAv vs WTv** | **Q Value** |
| --- | --- | --- | --- | --- |
| Gmppb | -12.0 | 0.023 | -5.0 | 0.001 |
| Ngp | -12.0 | 0.041 | 3.0 | 0.015 |
| Insm1 | -6.4 | 0.000 | 7.3 | 0.002 |
| Ppp1r3b | -5.2 | 0.002 | -4.2 | 0.011 |
| Rbm46 | -4.6 | 0.030 | 4.0 | 0.018 |
| Lamc2 | -4.5 | 0.040 | 5.3 | 0.002 |
| 1700003M02Rik | -4.1 | 0.024 | -3.5 | 0.001 |
| Stfa2 | -4.0 | 0.044 | 3.6 | 0.018 |
| Gm13293 | -4.0 | 0.014 | -17.0 | 0.047 |
| Rfc3 | -3.8 | 0.020 | -3.2 | 0.040 |
| Slc10a3 | -3.6 | 0.002 | 2.9 | 0.000 |
| Sgcd | -3.6 | 0.000 | 2.4 | 0.006 |
| Urb1 | -3.6 | 0.004 | 2.9 | 0.025 |
| Ifi30 | -3.5 | 0.039 | 3.1 | 0.016 |
| Spsb4 | -3.5 | 0.049 | -2.1 | 0.029 |
| 2210403K04Rik | -3.5 | 0.011 | -3.2 | 0.004 |
| Lsm5 | -3.2 | 0.001 | -1.7 | 0.046 |
| Shisa3 | -3.2 | 0.002 | 3.8 | 0.001 |
| Slit1 | -3.1 | 0.035 | -1.6 | 0.021 |
| Zic5 | -3.1 | 0.007 | 4.2 | 0.017 |
| Egfl7 | -2.9 | 0.000 | 1.3 | 0.000 |
| Ifltd1 | -2.9 | 0.016 | 5.1 | 0.003 |
| RP23-381B19.7 | -2.7 | 0.000 | -2.4 | 0.000 |
| Dcx | -2.7 | 0.000 | -1.2 | 0.003 |
| Ly6h | -2.6 | 0.020 | -2.9 | 0.002 |
| Chchd7 | -2.6 | 0.000 | -1.3 | 0.006 |
| Rnf213 | -2.5 | 0.020 | -3.1 | 0.018 |
| Cdon | -2.5 | 0.009 | -2.4 | 0.022 |
| Ddah2 | -2.4 | 0.000 | -2.0 | 0.003 |
| Gm11942 | -2.3 | 0.000 | -1.3 | 0.007 |
| Nol9 | -2.2 | 0.000 | -0.8 | 0.033 |
| Pih1d1 | -2.2 | 0.005 | -1.8 | 0.006 |
| Rplp1 | -2.2 | 0.000 | -1.2 | 0.012 |
| Lsm2 | -2.1 | 0.000 | -2.3 | 0.000 |
| Gm9385 | -2.1 | 0.003 | -1.6 | 0.006 |
| Hbb-b1 | -2.0 | 0.000 | -1.1 | 0.001 |
| Mrpl20 | -2.0 | 0.000 | -1.7 | 0.000 |
| Farsa | -2.0 | 0.016 | -1.1 | 0.038 |
| Amy1 | -1.9 | 0.017 | -3.2 | 0.000 |
| Rpl7 | -1.9 | 0.000 | -0.8 | 0.037 |
| Creb3 | -1.9 | 0.033 | -1.6 | 0.000 |
| Gm4997 | -1.9 | 0.001 | -1.5 | 0.023 |
| Rps20 | -1.8 | 0.000 | -1.2 | 0.008 |
| Lrrc42 | -1.8 | 0.047 | -1.5 | 0.040 |
| Myh14 | -1.8 | 0.000 | -1.4 | 0.042 |
| Maf1 | -1.8 | 0.004 | -1.8 | 0.011 |
| Rpl24 | -1.8 | 0.000 | -1.1 | 0.002 |
| Rps10 | -1.8 | 0.023 | -1.4 | 0.039 |
| Rps16 | -1.8 | 0.000 | -1.1 | 0.000 |
| Rps25 | -1.8 | 0.006 | -1.2 | 0.026 |
| Hn1 | -1.8 | 0.015 | -1.2 | 0.026 |
| Ogfr | -1.8 | 0.050 | -1.8 | 0.022 |
| Rpl32 | -1.8 | 0.001 | -1.3 | 0.003 |
| Rps15a | -1.8 | 0.001 | -1.2 | 0.018 |
| Zbtb8os | -1.8 | 0.032 | 1.2 | 0.024 |
| Rps25-ps1 | -1.7 | 0.000 | -1.2 | 0.002 |
| Rpl13 | -1.7 | 0.026 | -1.4 | 0.046 |
| Gm12918 | -1.7 | 0.000 | -1.4 | 0.000 |
| AC188461.4 | -1.7 | 0.049 | -2.0 | 0.001 |
| Rps16-ps2 | -1.7 | 0.000 | -1.1 | 0.000 |
| Rpl27 | -1.7 | 0.000 | -1.0 | 0.005 |
| Rpl19-ps1 | -1.7 | 0.000 | -1.1 | 0.002 |
| Rps4x | -1.7 | 0.000 | -0.9 | 0.000 |
| Gnb2l1 | -1.7 | 0.000 | -0.8 | 0.022 |
| Rps29 | -1.6 | 0.004 | -1.1 | 0.022 |
| Adcy3 | -1.6 | 0.016 | -1.5 | 0.004 |
| Gm8430 | -1.6 | 0.000 | -1.0 | 0.008 |
| Rnf130 | -1.6 | 0.008 | -1.0 | 0.001 |
| Igf2 | -1.5 | 0.000 | -1.6 | 0.003 |
| Znrd1 | -1.5 | 0.001 | -1.2 | 0.001 |
| Dctn3 | -1.5 | 0.020 | -1.0 | 0.027 |
| Npdc1 | -1.5 | 0.000 | -0.9 | 0.046 |
| Sqstm1 | -1.5 | 0.000 | -0.7 | 0.025 |
| Slc16a4 | -1.5 | 0.023 | -1.5 | 0.028 |
| Rpl11 | -1.5 | 0.000 | -1.0 | 0.011 |
| Resp18 | -1.5 | 0.000 | -1.4 | 0.000 |
| Romo1 | -1.5 | 0.000 | -1.0 | 0.000 |
| Gm1673 | -1.5 | 0.001 | -1.0 | 0.016 |
| Gm13826 | -1.5 | 0.000 | -1.2 | 0.000 |
| Calca | -1.4 | 0.036 | -1.7 | 0.001 |
| Rps9 | -1.4 | 0.000 | -0.5 | 0.020 |
| Gm11361 | -1.4 | 0.000 | -1.0 | 0.004 |
| Rpl23a | -1.4 | 0.000 | -1.4 | 0.000 |
| Atp5k | -1.4 | 0.003 | -0.8 | 0.024 |
| Rpl41 | -1.3 | 0.000 | -0.6 | 0.030 |
| Tmbim6 | -1.3 | 0.000 | -0.9 | 0.001 |
| Rpl7a | -1.3 | 0.039 | -1.2 | 0.032 |
| Tm2d1 | -1.3 | 0.004 | -1.9 | 0.000 |
| Rbmx | -1.3 | 0.018 | -1.1 | 0.029 |
| Rps24 | -1.3 | 0.000 | -1.1 | 0.000 |
| Sepw1 | -1.3 | 0.040 | -1.4 | 0.012 |
| Sdf2 | -1.3 | 0.045 | -1.0 | 0.001 |
| D4Wsu53e | -1.2 | 0.006 | -1.7 | 0.000 |
| BC004004 | -1.2 | 0.016 | 0.8 | 0.010 |
| Rps5 | -1.2 | 0.000 | -0.6 | 0.001 |
| Prkar2b | -1.2 | 0.000 | -1.0 | 0.002 |
| Rpl30 | -1.2 | 0.006 | -1.0 | 0.012 |
| Ndufb8 | -1.2 | 0.004 | -0.8 | 0.010 |
| 2010107E04Rik | -1.2 | 0.039 | -0.9 | 0.027 |
| 0610011F06Rik | -1.2 | 0.018 | -1.6 | 0.004 |
| Rpl23 | -1.2 | 0.000 | -0.8 | 0.000 |
| Btf3 | -1.2 | 0.000 | -0.8 | 0.000 |
| Pfdn5 | -1.2 | 0.000 | -0.7 | 0.007 |
| Susd2 | -1.2 | 0.006 | -1.5 | 0.000 |
| Gm1821 | -1.2 | 0.000 | -0.6 | 0.013 |
| Ubb | -1.2 | 0.000 | -0.7 | 0.002 |
| Ndufb10 | -1.2 | 0.019 | -1.3 | 0.000 |
| Ryr3 | -1.1 | 0.042 | -1.5 | 0.029 |
| Atp5g3 | -1.1 | 0.017 | -0.8 | 0.022 |
| Pdlim2 | -1.1 | 0.033 | -1.5 | 0.010 |
| Rps28 | -1.1 | 0.000 | -0.5 | 0.023 |
| Rps15 | -1.1 | 0.001 | -1.0 | 0.000 |
| Ndufa4 | -1.1 | 0.044 | -0.8 | 0.039 |
| Ubl5 | -1.1 | 0.010 | -0.8 | 0.019 |
| Commd3 | -1.1 | 0.014 | -1.0 | 0.005 |
| Prdx1 | -1.1 | 0.000 | -1.0 | 0.000 |
| Rabac1 | -1.0 | 0.000 | -0.9 | 0.000 |
| Uqcrq | -1.0 | 0.021 | -0.8 | 0.020 |
| Gpd1l | -1.0 | 0.017 | -1.3 | 0.000 |
| Srsf7 | -1.0 | 0.021 | 0.7 | 0.020 |
| Lmo4 | -1.0 | 0.000 | -0.5 | 0.000 |
| A730017C20Rik | -1.0 | 0.037 | 0.9 | 0.011 |
| 2700094K13Rik | -1.0 | 0.018 | -1.8 | 0.036 |
| Ppia | -0.9 | 0.002 | -0.6 | 0.006 |
| Atp6v0c | -0.9 | 0.000 | -0.6 | 0.016 |
| Tmsb4x | -0.9 | 0.000 | -0.8 | 0.000 |
| Gm13493 | -0.9 | 0.003 | -0.6 | 0.027 |
| Ublcp1 | -0.9 | 0.039 | -0.8 | 0.035 |
| Brp44l | -0.9 | 0.003 | -0.9 | 0.000 |
| Wbp5 | -0.9 | 0.015 | -1.3 | 0.000 |
| Eef1b2 | -0.7 | 0.000 | -0.4 | 0.010 |
| Sept15 | -0.7 | 0.000 | -0.7 | 0.000 |
| Atp1a1 | -0.6 | 0.004 | -0.4 | 0.017 |
| Sqle | -0.5 | 0.037 | -0.8 | 0.000 |
| Oaz2 | -0.4 | 0.020 | -0.8 | 0.000 |
| Aldoa | -0.3 | 0.006 | -0.6 | 0.000 |
| Polr2m | 0.4 | 0.015 | -0.3 | 0.001 |
| Spcs3 | 0.4 | 0.036 | 0.4 | 0.032 |
| Csde1 | 0.4 | 0.020 | -0.4 | 0.000 |
| Dctn4 | 0.4 | 0.021 | 0.2 | 0.020 |
| Rufy3 | 0.4 | 0.000 | -0.6 | 0.000 |
| Hnrnph1 | 0.6 | 0.023 | 0.9 | 0.000 |
| Smarca2 | 0.6 | 0.000 | 0.6 | 0.000 |
| Adam23 | 0.6 | 0.001 | -1.2 | 0.000 |
| Cdc42bpa | 0.6 | 0.030 | 0.3 | 0.039 |
| Psd3 | 0.6 | 0.041 | -0.5 | 0.027 |
| Zmynd11 | 0.7 | 0.019 | 1.0 | 0.008 |
| Hnrnpk | 0.7 | 0.000 | 0.5 | 0.000 |
| Mkl2 | 0.8 | 0.036 | 0.8 | 0.020 |
| Fgf11 | 0.8 | 0.022 | -0.6 | 0.000 |
| Zranb2 | 0.8 | 0.001 | 0.6 | 0.013 |
| Armcx3 | 0.8 | 0.000 | -0.4 | 0.036 |
| Pcm1 | 0.8 | 0.003 | -0.5 | 0.000 |
| Adam22 | 0.8 | 0.003 | -1.0 | 0.000 |
| 6430548M08Rik | 0.8 | 0.045 | -1.0 | 0.003 |
| Fam164a | 0.9 | 0.005 | 0.5 | 0.033 |
| Npm1 | 0.9 | 0.001 | 0.8 | 0.000 |
| Ppp5c | 0.9 | 0.021 | 1.2 | 0.000 |
| Anp32a | 0.9 | 0.018 | 1.0 | 0.011 |
| Ube3a | 0.9 | 0.003 | 0.4 | 0.002 |
| Specc1 | 1.0 | 0.000 | -0.6 | 0.000 |
| Rab11fip2 | 1.0 | 0.042 | 1.1 | 0.022 |
| Ssb | 1.0 | 0.001 | 0.8 | 0.001 |
| Gne | 1.0 | 0.017 | 1.2 | 0.000 |
| Supt6h | 1.0 | 0.019 | 0.7 | 0.022 |
| Sox2ot | 1.0 | 0.001 | -0.7 | 0.020 |
| Mpp2 | 1.0 | 0.049 | 1.1 | 0.047 |
| Fermt2 | 1.0 | 0.003 | 0.9 | 0.006 |
| Fam40b | 1.0 | 0.008 | -1.6 | 0.001 |
| Nupl1 | 1.0 | 0.016 | 0.7 | 0.009 |
| Zfp365 | 1.1 | 0.000 | 0.5 | 0.000 |
| Tmem176a | 1.1 | 0.042 | 0.9 | 0.000 |
| Cul4b | 1.1 | 0.002 | 0.6 | 0.010 |
| Sdcbp | 1.1 | 0.000 | 1.0 | 0.000 |
| Ext2 | 1.1 | 0.005 | 2.4 | 0.000 |
| Ube2d1 | 1.1 | 0.045 | -1.2 | 0.013 |
| Gmfb | 1.1 | 0.021 | 0.9 | 0.036 |
| Tjp1 | 1.1 | 0.000 | 0.6 | 0.006 |
| Eif4a2 | 1.1 | 0.000 | 0.9 | 0.000 |
| Luc7l3 | 1.1 | 0.000 | 0.8 | 0.006 |
| Peg3 | 1.1 | 0.002 | 1.0 | 0.001 |
| Samhd1 | 1.2 | 0.019 | 1.2 | 0.028 |
| Nrip3 | 1.2 | 0.010 | -1.0 | 0.041 |
| Ylpm1 | 1.2 | 0.021 | -1.0 | 0.005 |
| Ube2e2 | 1.2 | 0.000 | 1.2 | 0.000 |
| Mtus2 | 1.2 | 0.014 | -1.5 | 0.001 |
| Dnalc1 | 1.2 | 0.003 | -1.2 | 0.007 |
| Dnajc21 | 1.2 | 0.000 | 0.6 | 0.017 |
| Ipmk | 1.2 | 0.021 | 1.1 | 0.009 |
| Eps8 | 1.2 | 0.001 | 1.2 | 0.000 |
| Pdcd4 | 1.2 | 0.023 | 0.9 | 0.035 |
| Mtmr3 | 1.2 | 0.014 | -0.3 | 0.008 |
| Cpne1 | 1.2 | 0.018 | 2.2 | 0.000 |
| Golgb1 | 1.3 | 0.016 | 1.1 | 0.009 |
| Lztfl1 | 1.3 | 0.003 | 1.1 | 0.002 |
| Ppp3r1 | 1.3 | 0.012 | 0.9 | 0.017 |
| Mlh3 | 1.3 | 0.000 | -1.0 | 0.016 |
| Dnajb12 | 1.3 | 0.004 | 1.3 | 0.000 |
| Adra1a | 1.3 | 0.019 | -2.1 | 0.000 |
| Gm17312 | 1.3 | 0.046 | 1.2 | 0.022 |
| Cic | 1.3 | 0.012 | -1.5 | 0.001 |
| Pcgf1 | 1.4 | 0.028 | 1.4 | 0.012 |
| Slc29a3 | 1.4 | 0.001 | 0.9 | 0.001 |
| Uspl1 | 1.4 | 0.000 | 1.1 | 0.000 |
| 7SK | 1.4 | 0.034 | 2.8 | 0.000 |
| 7SK | 1.4 | 0.021 | 2.8 | 0.000 |
| 7SK | 1.4 | 0.043 | 2.9 | 0.000 |
| Mcart1 | 1.5 | 0.000 | 0.7 | 0.018 |
| Gm17555 | 1.5 | 0.048 | 1.4 | 0.016 |
| Gm17385 | 1.5 | 0.048 | 1.4 | 0.025 |
| Top1 | 1.5 | 0.002 | 1.2 | 0.004 |
| Trf | 1.5 | 0.000 | 0.4 | 0.018 |
| Cul3 | 1.5 | 0.000 | 0.7 | 0.027 |
| Gm17295 | 1.5 | 0.044 | 1.4 | 0.021 |
| Gm17316 | 1.5 | 0.045 | 1.4 | 0.021 |
| Mctp1 | 1.5 | 0.000 | -1.7 | 0.000 |
| Ube2q2 | 1.5 | 0.006 | -1.2 | 0.001 |
| Mir5102 | 1.6 | 0.034 | 1.0 | 0.046 |
| Gm17132 | 1.6 | 0.004 | 1.3 | 0.001 |
| Arrdc3 | 1.6 | 0.001 | 1.7 | 0.000 |
| Zfp385b | 1.6 | 0.000 | -1.3 | 0.017 |
| Dram2 | 1.6 | 0.000 | 1.1 | 0.000 |
| Alg6 | 1.7 | 0.006 | 1.5 | 0.025 |
| Gcnt2 | 1.7 | 0.003 | 2.3 | 0.044 |
| mt-Nd6 | 1.7 | 0.000 | 1.3 | 0.000 |
| Zfp353 | 1.7 | 0.012 | 1.3 | 0.020 |
| mt-Tl1 | 1.8 | 0.006 | 1.4 | 0.010 |
| R3hdm2 | 1.8 | 0.000 | -0.8 | 0.023 |
| Rnf111 | 1.8 | 0.000 | 1.3 | 0.012 |
| Dennd1b | 1.8 | 0.018 | 1.7 | 0.002 |
| Gm17386 | 1.8 | 0.001 | 1.8 | 0.000 |
| Slc6a5 | 1.9 | 0.000 | -0.9 | 0.036 |
| Prpf8 | 1.9 | 0.000 | 0.9 | 0.001 |
| Fnbp4 | 1.9 | 0.002 | -1.5 | 0.000 |
| Gfra2 | 1.9 | 0.001 | 1.6 | 0.000 |
| Slu7 | 1.9 | 0.000 | 1.3 | 0.021 |
| Gca | 1.9 | 0.020 | -1.3 | 0.026 |
| Mir5105 | 1.9 | 0.000 | 1.8 | 0.000 |
| Kcnn2 | 1.9 | 0.001 | 1.4 | 0.006 |
| Smpd4 | 2.0 | 0.000 | 1.5 | 0.000 |
| AA474408 | 2.0 | 0.000 | 1.9 | 0.000 |
| Cd97 | 2.1 | 0.006 | -1.2 | 0.043 |
| Fmn1 | 2.1 | 0.013 | 2.5 | 0.039 |
| Wdr35 | 2.1 | 0.000 | 1.4 | 0.001 |
| Trmt11 | 2.1 | 0.034 | -3.6 | 0.012 |
| Dnajb4 | 2.1 | 0.000 | 1.4 | 0.000 |
| Nupl2 | 2.2 | 0.018 | -2.0 | 0.032 |
| Etv6 | 2.2 | 0.004 | 1.5 | 0.034 |
| Esf1 | 2.2 | 0.000 | 1.0 | 0.042 |
| Gm11407 | 2.2 | 0.000 | 0.9 | 0.034 |
| Lars2 | 2.3 | 0.000 | 1.9 | 0.000 |
| mmu-mir-2134-4 | 2.3 | 0.000 | 1.3 | 0.001 |
| mmu-mir-689-2 | 2.4 | 0.000 | 1.3 | 0.000 |
| mmu-mir-2134-4 | 2.4 | 0.000 | 1.3 | 0.000 |
| mmu-mir-2134-2 | 2.4 | 0.000 | 1.3 | 0.000 |
| Cachd1 | 2.7 | 0.000 | -1.7 | 0.004 |
| Filip1 | 2.7 | 0.011 | -4.3 | 0.000 |
| Cdkn1a | 2.8 | 0.000 | 4.8 | 0.000 |
| Gm13845 | 2.8 | 0.016 | -3.5 | 0.019 |
| mt-Nd4l | 2.8 | 0.000 | 1.5 | 0.000 |
| Gm10222 | 2.9 | 0.000 | 1.4 | 0.000 |
| Zfp449 | 2.9 | 0.002 | 2.0 | 0.050 |
| C330007P06Rik | 2.9 | 0.000 | 1.5 | 0.001 |
| Gm15564 | 3.1 | 0.000 | 2.5 | 0.000 |
| Hsf4 | 3.4 | 0.035 | 2.0 | 0.011 |
| Jph1 | 3.6 | 0.004 | -3.4 | 0.018 |
| Lrdd | 3.7 | 0.024 | 4.7 | 0.001 |
| Snord118 | 3.8 | 0.045 | 4.6 | 0.003 |
| 2210406O10Rik | 4.0 | 0.001 | -5.0 | 0.000 |
| Slc2a9 | 5.4 | 0.005 | 3.7 | 0.002 |
| Hrh2 | 5.5 | 0.000 | -3.7 | 0.033 |
| Gm9934 | 5.8 | 0.000 | 4.3 | 0.011 |
| Ccdc135 | 7.2 | 0.001 | 3.5 | 0.018 |
