# Supplementary material for: Transcriptional profiling of differentially vulnerable motor neurons at pre-symptomatic stage in the Smn2b/- mouse model of spinal muscular atrophy
Source: Acta Neuropathol Commun. 2015 Sep 15;3:55. doi: 10.1186/s40478-015-0231-1 (PMC4570693; doi:10.1186/s40478-015-0231-1)
Supplement: Additional file 3: Table S3. — Table show a list of genes which are differentially expressed between SMAv motor neurons when compared to SMAr or Wtv motor neurons. (DOCX 113 kb) [file 40478_2015_231_MOESM3_ESM.docx]

**Supplementary Table 3: Statistically altered transcriptional changes in SMAv motor neurons compared to WTv and SMAr motor neurons.**

| **Official Gene Symbol** | **SMAv vs WTv** | **Q Value** | **SMAv vs SMAr** | **Q Value** |
| --- | --- | --- | --- | --- |
| Aebp1 | -17.0 | 0.027 | -18.5 | 0.011 |
| Zic3 | -6.3 | 0.000 | -5.1 | 0.010 |
| Reck | -6.2 | 0.011 | -5.8 | 0.026 |
| Ccnb2 | -5.1 | 0.000 | -5.4 | 0.000 |
| Gm10684 | -5.0 | 0.020 | -5.4 | 0.017 |
| 2210406O10Rik | -5.0 | 0.000 | -4.2 | 0.001 |
| Camkk2 | -4.8 | 0.003 | -4.5 | 0.012 |
| Myo7a | -4.4 | 0.006 | -4.6 | 0.005 |
| Filip1 | -4.3 | 0.000 | -5.3 | 0.000 |
| Emr1 | -4.2 | 0.026 | -4.5 | 0.027 |
| Nr6a1 | -4.1 | 0.013 | -3.5 | 0.034 |
| 4930581F22Rik | -3.9 | 0.029 | -4.4 | 0.016 |
| 1700030K09Rik | -3.9 | 0.040 | -5.3 | 0.004 |
| Hrh2 | -3.7 | 0.033 | -6.4 | 0.000 |
| Bex4 | -3.7 | 0.011 | -3.9 | 0.015 |
| Trmt11 | -3.6 | 0.012 | -3.6 | 0.018 |
| Gm13845 | -3.5 | 0.019 | -6.3 | 0.000 |
| A330049M08Rik | -3.5 | 0.019 | -3.7 | 0.026 |
| Igf1 | -3.5 | 0.000 | -4.9 | 0.000 |
| Myo5b | -3.5 | 0.004 | -3.7 | 0.007 |
| Gda | -3.5 | 0.010 | -3.7 | 0.011 |
| D330045A20Rik | -3.4 | 0.014 | -3.9 | 0.024 |
| Jph1 | -3.4 | 0.018 | -5.1 | 0.000 |
| Sirt7 | -3.4 | 0.000 | -3.0 | 0.007 |
| Klhdc1 | -3.3 | 0.047 | -3.6 | 0.007 |
| Zic1 | -3.1 | 0.046 | -7.9 | 0.000 |
| Eya1 | -3.0 | 0.016 | -3.1 | 0.024 |
| Ddx3y | -3.0 | 0.000 | -2.2 | 0.023 |
| Mxra8 | -2.9 | 0.010 | -3.8 | 0.002 |
| Dhrs7 | -2.9 | 0.031 | -3.0 | 0.048 |
| Col4a5 | -2.8 | 0.017 | -3.0 | 0.012 |
| Hoxd3 | -2.8 | 0.024 | -3.7 | 0.009 |
| Dmrta1 | -2.6 | 0.049 | -3.7 | 0.019 |
| Vars2 | -2.4 | 0.000 | -2.2 | 0.018 |
| Yrdc | -2.3 | 0.001 | -2.8 | 0.000 |
| Abhd16a | -2.3 | 0.000 | -1.9 | 0.008 |
| Wipf3 | -2.3 | 0.005 | -2.2 | 0.002 |
| Mum1l1 | -2.3 | 0.000 | -2.3 | 0.001 |
| Pus3 | -2.3 | 0.017 | -1.8 | 0.049 |
| C230052I12Rik | -2.2 | 0.000 | -1.7 | 0.025 |
| Pex10 | -2.2 | 0.016 | -2.1 | 0.049 |
| Myl9 | -2.2 | 0.021 | -2.6 | 0.002 |
| P4ha3 | -2.2 | 0.014 | -4.0 | 0.000 |
| Man2c1 | -2.1 | 0.012 | -2.4 | 0.022 |
| Adra1a | -2.1 | 0.000 | -1.3 | 0.033 |
| Rfx4 | -2.0 | 0.004 | -2.1 | 0.004 |
| Tank | -2.0 | 0.000 | -1.7 | 0.001 |
| Nupl2 | -2.0 | 0.032 | -3.4 | 0.000 |
| Pou2f1 | -2.0 | 0.016 | -1.9 | 0.009 |
| Donson | -2.0 | 0.040 | -3.0 | 0.000 |
| Srebf1 | -1.8 | 0.000 | -1.9 | 0.015 |
| Bmpr1b | -1.8 | 0.015 | -2.4 | 0.004 |
| Maf | -1.8 | 0.036 | -2.1 | 0.017 |
| A530058N18Rik | -1.8 | 0.024 | -2.3 | 0.011 |
| Cachd1 | -1.7 | 0.004 | -2.8 | 0.000 |
| Ctsh | -1.7 | 0.034 | -2.6 | 0.000 |
| Fign | -1.7 | 0.030 | -2.9 | 0.000 |
| B3galt2 | -1.7 | 0.025 | -1.8 | 0.029 |
| Pttg1ip | -1.7 | 0.003 | -1.5 | 0.031 |
| Mctp1 | -1.7 | 0.000 | -1.4 | 0.016 |
| Ppp1r18 | -1.6 | 0.012 | -1.4 | 0.045 |
| Zfyve28 | -1.6 | 0.011 | -2.3 | 0.001 |
| Tmem64 | -1.6 | 0.042 | -2.3 | 0.002 |
| Fam40b | -1.6 | 0.001 | -1.6 | 0.011 |
| Igf2 | -1.6 | 0.003 | -4.2 | 0.000 |
| Rps6kb2 | -1.5 | 0.043 | -1.7 | 0.008 |
| Pard3 | -1.5 | 0.005 | -1.9 | 0.004 |
| Mtus2 | -1.5 | 0.001 | -1.4 | 0.002 |
| Chd3 | -1.5 | 0.000 | -1.8 | 0.000 |
| Fnbp4 | -1.5 | 0.000 | -1.5 | 0.003 |
| Cic | -1.5 | 0.001 | -2.0 | 0.000 |
| 2610203C20Rik | -1.5 | 0.023 | -1.8 | 0.007 |
| Pus7 | -1.5 | 0.043 | -2.1 | 0.004 |
| Atp13a5 | -1.5 | 0.035 | -2.0 | 0.007 |
| Nsg2 | -1.4 | 0.019 | -1.4 | 0.049 |
| Tmem62 | -1.4 | 0.018 | -1.7 | 0.004 |
| Efr3b | -1.4 | 0.000 | -1.0 | 0.009 |
| Dnajc1 | -1.4 | 0.029 | -2.1 | 0.000 |
| Zfp259 | -1.3 | 0.022 | -1.8 | 0.003 |
| Rit1 | -1.3 | 0.001 | -2.0 | 0.000 |
| Wbp5 | -1.3 | 0.000 | -1.2 | 0.005 |
| Chchd7 | -1.3 | 0.006 | 1.8 | 0.013 |
| Rbms2 | -1.3 | 0.029 | -2.0 | 0.000 |
| Trpc1 | -1.3 | 0.023 | -1.7 | 0.049 |
| 1810014F10Rik | -1.3 | 0.000 | -1.0 | 0.028 |
| Zfp609 | -1.2 | 0.005 | -1.6 | 0.000 |
| Gpr85 | -1.2 | 0.004 | -1.3 | 0.047 |
| Ube2q2 | -1.2 | 0.001 | -1.6 | 0.000 |
| Dnalc1 | -1.2 | 0.007 | -1.5 | 0.001 |
| Vps52 | -1.2 | 0.024 | -1.6 | 0.011 |
| Cd97 | -1.2 | 0.043 | -1.4 | 0.011 |
| Ubtf | -1.2 | 0.034 | -1.5 | 0.028 |
| Ftsjd2 | -1.2 | 0.000 | -1.1 | 0.031 |
| Nrxn2 | -1.2 | 0.011 | -1.0 | 0.047 |
| Ube2d1 | -1.2 | 0.013 | -1.7 | 0.000 |
| Chd8 | -1.2 | 0.001 | -1.1 | 0.007 |
| Kdm1b | -1.2 | 0.027 | -1.6 | 0.011 |
| Tmem8b | -1.1 | 0.002 | -1.4 | 0.045 |
| Bre | -1.1 | 0.000 | -1.2 | 0.002 |
| Zfp280d | -1.1 | 0.004 | -1.9 | 0.000 |
| Guf1 | -1.1 | 0.002 | -1.2 | 0.002 |
| Rfk | -1.1 | 0.000 | -0.9 | 0.002 |
| Tmem127 | -1.1 | 0.001 | -1.0 | 0.026 |
| Sephs1 | -1.1 | 0.034 | -1.5 | 0.005 |
| Tgfb1i1 | -1.1 | 0.007 | -1.6 | 0.003 |
| Hbb-b1 | -1.1 | 0.001 | -1.7 | 0.000 |
| Mlh3 | -1.0 | 0.016 | -1.3 | 0.000 |
| 6430548M08Rik | -1.0 | 0.003 | -1.6 | 0.000 |
| Adam22 | -1.0 | 0.000 | -0.7 | 0.002 |
| Ylpm1 | -1.0 | 0.005 | -1.3 | 0.005 |
| Pitrm1 | -1.0 | 0.007 | -0.9 | 0.017 |
| Edil3 | -1.0 | 0.000 | -0.9 | 0.000 |
| Mll5 | -0.9 | 0.036 | -1.1 | 0.013 |
| Gnal | -0.9 | 0.017 | -1.0 | 0.025 |
| S100pbp | -0.9 | 0.047 | -1.5 | 0.001 |
| Dbi | -0.9 | 0.000 | -0.8 | 0.000 |
| Nr2c2 | -0.9 | 0.033 | -1.8 | 0.000 |
| Hoxa7 | -0.8 | 0.006 | 6.7 | 0.000 |
| Eif4g3 | -0.8 | 0.001 | -0.5 | 0.010 |
| R3hdm2 | -0.8 | 0.023 | -1.2 | 0.000 |
| Msi2 | -0.8 | 0.013 | -1.5 | 0.000 |
| Ugp2 | -0.8 | 0.006 | -0.7 | 0.017 |
| Yaf2 | -0.8 | 0.041 | -1.0 | 0.004 |
| Sh3kbp1 | -0.8 | 0.027 | -1.7 | 0.000 |
| Rian | -0.7 | 0.015 | -0.6 | 0.039 |
| Rps14 | -0.7 | 0.000 | 0.7 | 0.030 |
| Sqstm1 | -0.7 | 0.025 | 1.2 | 0.001 |
| Igfbp5 | -0.7 | 0.038 | -2.6 | 0.000 |
| Dclk1 | -0.6 | 0.002 | -0.5 | 0.034 |
| Cdh18 | -0.6 | 0.016 | -1.1 | 0.001 |
| Ybx1 | -0.6 | 0.010 | -0.7 | 0.005 |
| Specc1 | -0.6 | 0.000 | -0.8 | 0.000 |
| Rufy3 | -0.6 | 0.000 | -0.4 | 0.004 |
| Arpp21 | -0.6 | 0.047 | -1.8 | 0.000 |
| Arhgap23 | -0.6 | 0.008 | -0.9 | 0.000 |
| Zfp207 | -0.6 | 0.048 | -0.7 | 0.038 |
| Psd3 | -0.5 | 0.027 | -0.8 | 0.000 |
| Pcdhga9 | -0.5 | 0.011 | -1.0 | 0.000 |
| Zmiz2 | -0.5 | 0.000 | -1.0 | 0.000 |
| Pcm1 | -0.5 | 0.000 | -0.7 | 0.000 |
| Cnot4 | -0.4 | 0.002 | -0.8 | 0.000 |
| Armcx3 | -0.4 | 0.036 | -0.7 | 0.000 |
| Pak1 | -0.3 | 0.007 | -0.4 | 0.000 |
| Polr2m | -0.3 | 0.001 | -0.7 | 0.000 |
| Pnkd | -0.3 | 0.028 | 0.4 | 0.034 |
| Cdc42bpa | 0.3 | 0.039 | -0.4 | 0.034 |
| Ube3a | 0.4 | 0.002 | -0.3 | 0.028 |
| Esrrg | 0.6 | 0.001 | -0.7 | 0.016 |
| 3632451O06Rik | 0.6 | 0.017 | 0.6 | 0.037 |
| Smarca2 | 0.6 | 0.000 | -0.3 | 0.002 |
| Tjp1 | 0.6 | 0.006 | -0.8 | 0.006 |
| Ddb1 | 0.6 | 0.004 | 0.6 | 0.007 |
| Mosc2 | 0.7 | 0.023 | 0.9 | 0.038 |
| Cript | 0.7 | 0.013 | 1.2 | 0.000 |
| Ablim2 | 0.7 | 0.006 | 0.8 | 0.015 |
| Srsf7 | 0.7 | 0.020 | 0.9 | 0.023 |
| Mrpl37 | 0.8 | 0.037 | 1.1 | 0.004 |
| Suds3 | 0.8 | 0.027 | 1.2 | 0.002 |
| BC004004 | 0.8 | 0.010 | 1.3 | 0.001 |
| Moap1 | 0.9 | 0.035 | 1.4 | 0.011 |
| Prpf8 | 0.9 | 0.001 | -0.9 | 0.006 |
| Cald1 | 0.9 | 0.000 | -0.8 | 0.011 |
| Gm11407 | 0.9 | 0.034 | -1.1 | 0.012 |
| Mysm1 | 1.0 | 0.015 | 1.5 | 0.001 |
| Tspan12 | 1.0 | 0.014 | 1.2 | 0.028 |
| Mfn1 | 1.0 | 0.014 | 1.4 | 0.004 |
| Hk1 | 1.0 | 0.001 | 0.9 | 0.031 |
| Acsl5 | 1.1 | 0.023 | 2.6 | 0.000 |
| Ttc1 | 1.1 | 0.001 | 1.0 | 0.022 |
| Map3k7 | 1.1 | 0.001 | 1.1 | 0.012 |
| Arhgef2 | 1.1 | 0.004 | 1.2 | 0.016 |
| Idh1 | 1.1 | 0.000 | 0.6 | 0.023 |
| Rabggtb | 1.1 | 0.000 | 0.6 | 0.028 |
| Eme2 | 1.2 | 0.006 | 1.9 | 0.001 |
| Zbtb8os | 1.2 | 0.024 | 2.0 | 0.005 |
| Lass5 | 1.2 | 0.012 | 1.2 | 0.028 |
| Nr1d1 | 1.2 | 0.007 | 1.4 | 0.047 |
| Slc36a1 | 1.2 | 0.043 | 1.8 | 0.007 |
| Rbm43 | 1.3 | 0.000 | 1.8 | 0.001 |
| Col1a1 | 1.3 | 0.039 | -2.2 | 0.000 |
| Fbxl18 | 1.3 | 0.036 | 1.5 | 0.023 |
| Jmjd6 | 1.3 | 0.004 | 1.6 | 0.008 |
| Gpr19 | 1.3 | 0.004 | 1.5 | 0.045 |
| Egfl7 | 1.3 | 0.000 | 4.4 | 0.000 |
| Traf4 | 1.3 | 0.011 | 1.5 | 0.045 |
| Car4 | 1.4 | 0.001 | 1.6 | 0.010 |
| Sfi1 | 1.4 | 0.018 | 1.7 | 0.020 |
| Agpat3 | 1.4 | 0.000 | 1.2 | 0.001 |
| Tmem39a | 1.4 | 0.002 | 1.3 | 0.018 |
| Gm10222 | 1.4 | 0.000 | -1.3 | 0.000 |
| Rad52 | 1.5 | 0.002 | 1.7 | 0.002 |
| Mfhas1 | 1.5 | 0.017 | 1.8 | 0.023 |
| Gpn1 | 1.5 | 0.023 | 2.2 | 0.019 |
| mt-Nd4l | 1.5 | 0.000 | -1.3 | 0.000 |
| Wdr75 | 1.6 | 0.000 | 1.6 | 0.007 |
| Stard3 | 1.6 | 0.003 | 3.2 | 0.004 |
| Stt3a | 1.7 | 0.000 | 1.5 | 0.001 |
| Utp14a | 1.7 | 0.003 | 1.5 | 0.044 |
| Apba2 | 1.7 | 0.000 | 1.1 | 0.013 |
| Prpf3 | 1.8 | 0.000 | 2.2 | 0.005 |
| Crtc3 | 1.8 | 0.042 | 2.4 | 0.019 |
| Dtx2 | 2.0 | 0.004 | 2.8 | 0.008 |
| Gm17427 | 2.0 | 0.026 | 5.4 | 0.012 |
| Ctnna3 | 2.0 | 0.000 | 2.8 | 0.000 |
| Angel1 | 2.1 | 0.000 | 2.6 | 0.008 |
| Insig2 | 2.1 | 0.000 | 1.7 | 0.003 |
| Pax2 | 2.1 | 0.050 | 3.1 | 0.019 |
| Zfp354b | 2.1 | 0.035 | 3.7 | 0.001 |
| Bola1 | 2.1 | 0.011 | 2.8 | 0.042 |
| 9030617O03Rik | 2.2 | 0.000 | 1.7 | 0.035 |
| Dnajc25 | 2.2 | 0.006 | 2.4 | 0.022 |
| 4930480K23Rik | 2.2 | 0.042 | 4.3 | 0.004 |
| Sun1 | 2.2 | 0.000 | 2.1 | 0.005 |
| Gm7120 | 2.3 | 0.001 | 2.3 | 0.036 |
| Trex1 | 2.4 | 0.020 | 5.7 | 0.004 |
| 1110021J02Rik | 2.4 | 0.001 | 2.4 | 0.014 |
| Sgcd | 2.4 | 0.006 | 4.2 | 0.000 |
| Rrm2 | 2.4 | 0.007 | 3.1 | 0.009 |
| Zbtb7b | 2.4 | 0.000 | 2.4 | 0.014 |
| Ext2 | 2.4 | 0.000 | -1.2 | 0.003 |
| 1700007K13Rik | 2.5 | 0.001 | 2.0 | 0.034 |
| Samd12 | 2.6 | 0.004 | 2.0 | 0.045 |
| Wdr96 | 2.6 | 0.001 | 13.0 | 0.000 |
| Slc27a3 | 2.6 | 0.000 | 4.4 | 0.004 |
| Sept1 | 2.7 | 0.026 | 13.0 | 0.003 |
| Ccdc99 | 2.7 | 0.049 | 13.0 | 0.039 |
| Cnot6l | 2.7 | 0.000 | 1.5 | 0.005 |
| Gm17652 | 2.8 | 0.017 | 3.4 | 0.018 |
| Arhgap33 | 2.9 | 0.000 | 2.3 | 0.008 |
| Bspry | 2.9 | 0.008 | 13.0 | 0.031 |
| Sstr1 | 2.9 | 0.005 | 4.5 | 0.016 |
| Slc10a3 | 2.9 | 0.000 | 3.6 | 0.001 |
| Dsn1 | 3.0 | 0.002 | 5.2 | 0.000 |
| Gm53 | 3.0 | 0.027 | 5.6 | 0.031 |
| Phf20 | 3.0 | 0.000 | 2.4 | 0.000 |
| 9330101J02Rik | 3.1 | 0.002 | 3.0 | 0.006 |
| Ifi30 | 3.1 | 0.016 | 3.5 | 0.048 |
| Acbd4 | 3.1 | 0.002 | 2.3 | 0.020 |
| Nlrp4f | 3.2 | 0.041 | 3.9 | 0.025 |
| Gm10047 | 3.2 | 0.001 | 3.0 | 0.027 |
| Dcxr | 3.3 | 0.000 | 3.0 | 0.005 |
| 4930547M16Rik | 3.3 | 0.013 | 4.3 | 0.003 |
| Cdc42ep4 | 3.4 | 0.000 | 3.1 | 0.000 |
| 4930441O14Rik | 3.4 | 0.008 | 3.7 | 0.023 |
| Bahcc1 | 3.4 | 0.005 | 5.5 | 0.002 |
| Chrnb1 | 3.4 | 0.020 | 4.2 | 0.006 |
| Gm15767 | 3.5 | 0.039 | 4.9 | 0.037 |
| Itpripl1 | 3.6 | 0.001 | 3.4 | 0.021 |
| Slc24a4 | 3.6 | 0.028 | 4.0 | 0.029 |
| Rassf6 | 3.6 | 0.025 | 3.8 | 0.024 |
| Stfa2 | 3.6 | 0.018 | 4.1 | 0.042 |
| Ano1 | 3.6 | 0.001 | 3.0 | 0.029 |
| Ccdc9 | 3.7 | 0.000 | 3.5 | 0.000 |
| 1700024P16Rik | 3.8 | 0.001 | 5.4 | 0.001 |
| Gm166 | 3.8 | 0.017 | 5.5 | 0.010 |
| Shisa3 | 3.8 | 0.001 | 2.6 | 0.043 |
| Gm10621 | 3.8 | 0.015 | 5.2 | 0.001 |
| 5330416C01Rik | 3.9 | 0.046 | 3.8 | 0.040 |
| Rbm46 | 4.0 | 0.018 | 4.4 | 0.037 |
| Hsf5 | 4.0 | 0.015 | 3.8 | 0.020 |
| Ovol1 | 4.1 | 0.011 | 4.4 | 0.034 |
| Gm16983 | 4.2 | 0.000 | 5.7 | 0.001 |
| Cbx7 | 4.3 | 0.001 | 3.6 | 0.002 |
| Gm13830 | 4.3 | 0.004 | 5.0 | 0.003 |
| Tbx20 | 4.4 | 0.016 | -3.0 | 0.032 |
| Crb1 | 4.4 | 0.000 | 2.5 | 0.046 |
| Impg2 | 4.5 | 0.007 | 4.8 | 0.019 |
| Trim34a | 4.6 | 0.000 | 3.2 | 0.006 |
| Pdia5 | 4.7 | 0.004 | 4.4 | 0.005 |
| Arntl2 | 4.7 | 0.000 | 3.2 | 0.012 |
| Chst8 | 4.7 | 0.000 | 5.2 | 0.000 |
| Cdkn1a | 4.8 | 0.000 | 1.5 | 0.000 |
| Gm11581 | 5.0 | 0.001 | 4.0 | 0.027 |
| I830134H01Rik | 5.1 | 0.010 | 5.4 | 0.037 |
| Pou2f2 | 5.2 | 0.000 | 2.5 | 0.018 |
| Lamc2 | 5.3 | 0.002 | 5.4 | 0.011 |
| Gm12811 | 5.3 | 0.016 | 5.2 | 0.007 |
| Txndc2 | 5.3 | 0.016 | 4.1 | 0.022 |
| 4933406C10Rik | 5.6 | 0.000 | 5.2 | 0.001 |
| Egflam | 5.7 | 0.000 | 3.4 | 0.006 |
| Zfp750 | 5.7 | 0.000 | 3.4 | 0.043 |
| Serpinb5 | 5.7 | 0.001 | 3.4 | 0.009 |
| Pmaip1 | 5.9 | 0.000 | 5.2 | 0.000 |
| Gm4793 | 5.9 | 0.000 | 4.5 | 0.009 |
| Gm11650 | 6.2 | 0.000 | 6.1 | 0.000 |
| Gm16704 | 6.2 | 0.000 | 3.3 | 0.031 |
| Tgif1 | 6.4 | 0.006 | 4.8 | 0.002 |
| Gm3294 | 6.4 | 0.039 | 5.2 | 0.007 |
| Plek2 | 7.1 | 0.018 | 3.9 | 0.015 |
| Insm1 | 7.3 | 0.002 | 5.1 | 0.000 |
| Gm10787 | 18.0 | 0.041 | 13.0 | 0.041 |
| Tmem29 | 18.0 | 0.043 | 13.0 | 0.043 |
